# ﻿Biogeographic factors contributing to the diversification of Euphoniinae (Aves, Passeriformes, Fringillidae): a phylogenetic and ancestral areas analysis

**DOI:** 10.3897/zookeys.1188.107047

**Published:** 2024-01-08

**Authors:** Melisa Vázquez-López, Sandra M. Ramírez-Barrera, Alondra K. Terrones-Ramírez, Sahid M. Robles-Bello, Adrián Nieto-Montes de Oca, Kristen Ruegg, Blanca E. Hernández-Baños

**Affiliations:** 1 Museo de Zoología, Departamento de Biología Evolutiva, Facultad de Ciencias, Universidad Nacional Autónoma de México, Ciudad de México, Mexico; 2 Posgrado en Ciencias Biológicas, Universidad Nacional Autónoma de México, Ciudad de México, Mexico; 3 Colorado State University, Fort Collins, Colorado, USA

**Keywords:** Atlantic Forest, Caribbean, diversification, Euphoniinae, Isthmus of Panama, Neotropical, trans-cis Andean areas

## Abstract

Factors such as the Andean uplift, Isthmus of Panama, and climate changes have influenced bird diversity in the Neotropical region. Studying bird species that are widespread in Neotropical highlands and lowlands can help us understand the impact of these factors on taxa diversification. Our main objectives were to determine the biogeographic factors that contributed to the diversification of Euphoniinae and re-evaluate their phylogenetic relationships. The nextRAD and mitochondrial data were utilized to construct phylogenies. The ancestral distribution range was then estimated using a time-calibrated phylogeny, current species ranges, and neotropical regionalization. The phylogenies revealed two main Euphoniinae clades, *Chlorophonia* and *Euphonia*, similar to previous findings. Furthermore, each genus has distinctive subclades corresponding to morphology and geography. The biogeographic results suggest that the Andean uplift and the establishment of the western Amazon drove the vicariance of *Chlorophonia* and *Euphonia* during the Miocene. The *Chlorophonia* lineage originated in the Andes mountains and spread to Central America and the Mesoamerican highlands after the formation of the Isthmus of Panama. Meanwhile, the ancestral area of *Euphonia* was the Amazonas, from which it spread to trans-Andean areas during the Pliocene and Pleistocene due to the separation of the west lowlands from Amazonas due to the Northern Andean uplift. *Chlorophonia* and *Euphonia* species migrated to the Atlantic Forest during the Pleistocene through corridors from the East Andean Humid Forest and Amazonas. These two genera had Caribbean invasions with distinct geographic origins and ages. Finally, we suggested taxonomic changes in the genus *Euphonia* based on the study’s phylogenetic, morphological, and biogeographic findings.

## ﻿Introduction

The Neotropical region is known for its significant diversity, which results from a combination of events including the Andean uplift, the formation of the Isthmus of Panama, changes in the Amazon basin and riverine landscape, and variations in vegetation biomes due to climatic oscillations and geologic events (Mittermeier et al. 2005; Hoorn et al. 2010; Smith and Klicka 2010; Ribas et al. 2012; Fox et al. 2015; Capurucho et al. 2018; Carneiro et al. 2018; Thode et al. 2019; Méndez-Camacho et al. 2021). Studies on biogeography and phylogeny have helped to identify the key drivers of biodiversity in the region. For instance, research on the phylogenetic relationships of widespread Neotropical avian lineages has revealed that biogeographic patterns may have resulted from multiple historical events at different periods (Smith et al. 2014), which have impacted highland and lowland species differently.

The Andean uplift had a significant impact on the diversification of both highland and lowland avifauna. One example of how the Andes Mountain range has impacted avifauna diversification is through the gradual uplift chain. This process resulted in speciation as species dispersed from lowlands to different altitudes (Ribas et al. 2007). Additionally, vicariant speciation occurred along the separated mountain blocks and changes in highland biomes due the climatic shifts (Gutiérrez-Zuluaga et al. 2021). Another way in which the Andean uplift influenced the avifauna is through the Miocene-Pliocene Northern uplift pulses. These pulses transformed the landscape of the northwestern Amazonas areas, creating a humid forest where there was once a wetland. This event was responsible for many Amazonas bird lineages originating in Western Amazonas (Carneiro et al. 2018). The Andean chain also separated avian lineages of the Pacific lowland forest from those of western Amazonas during the Pliocene. The impact of the Andean uplift on these biomes has also been observed in younger avian lineages (Reyes et al. 2023). In the lowlands, the Dry Diagonal also created a divide between the East Areas of the Amazonas, resulting in the formation of the Atlantic Forest, a biome with various endemic bird species. Biogeographic and phylogenetic research indicates that the diversification in this region may have been impacted by historical cycles of moist forest corridors in different parts of the Dry Diagonal that connected the Amazonas and the East Andean Forests with the Atlantic Forest (Capurucho et al. 2018; Trujillo-Arias et al. 2018). The Isthmus of Panama also played a role in the diversity of bird species in both the highlands and lowlands, facilitating the exchange of taxa in northward and southward directions across the Isthmus (Antonelli et al. 2010; Smith and Klicka 2010).

Phylogenetic and biogeographic studies on Neotropical birds that are widespread in highland and lowland tropical forests can provide insight into the drivers of biodiversity in lineages that inhabit the main Neotropical biomes. An interesting model taxon is the subfamily Euphoniinae, which belongs to the cosmopolitan family Fringillidae and is an extensive Neotropical endemic lineage (Zuccon et al. 2012). The subfamily consists of two genera, *Chlorophonia* (Bonaparte, 1851) and *Euphonia* (Desmarest, 1806), according to the current classification by the American Ornithological Society (Chesser et al. 2021). The first phylogenetic and biogeographic revision for this group was published just three years ago (Imfeld et al. 2020), which was significant for understanding Euphoniinae diversity in three different contexts: phylogenetic relationships, taxonomic implications, and biogeographic history. The study confirmed the paraphyly of the genus *Euphonia*, with the blue-headed Euphonias being the sister group of the *Chlorophonia* (Imfeld et al. 2020), based on this they formally resurrected the genus *Cyanophonia* (Bonaparte, 1851). However, the paraphyly was resolved by included these species into *Chlorophonia* genus (Chesser et al. 2021). This work also revealed that the genus *Euphonia* represents the most speciose and oldest group in the subfamily (Imfeld et al. 2020), with five clades that partially agree with the previous morphological grouping (Isler and Isler 1999). Biogeographic analysis indicated that the ancestor of Euphoniinae arrived in South America from the Eastern Hemisphere via a transoceanic route (13.8–7.1 Mya), and then diversified in South America (Imfeld et al. 2020). The biogeographic patterns for the two Euphoniinae genera are different. The blue-headed *Chlorophonia* were widespread throughout North and South America until 1.8 Mya, and species diversification in this group could be the result of vicariant speciation drivers by Pleistocene climate cycles that isolated highland Neotropical forests (Imfeld et al. 2020). Meanwhile, in *Euphonia* and the rest of *Chlorophonia*, the older lineages evolved in South America, and the adaptations to the tropical environment implied northward migrations until the Isthmus of Panama was entirely formed, after which the tropical forest expanded to North America (Imfeld et al. 2020). According to the authors, these migrations to new areas resulted in new lineage diversification in both genera and added evidence that the Isthmus of Panama could have been completely closed during the Pliocene (Imfeld et al. 2020). The study also identified two independent Caribbean migrations from South America by island-hopping and over-water dispersal (Imfeld et al. 2020).

Despite this remarkable knowledge on Euphoniinae diversification, there are still unanswered questions. While it is suggested that the Euphoniinae lineage reached South America via a transoceanic route, doubt has been cast on this explanation by biogeographic evidence that indicates that deep lineages of Fringillidae could have originated in North America at an earlier stage (Oliveros et al. 2019), and the deepest nodes of other Fringillidae subfamilies have Palearctic distributions (Zuccon et al. 2012; Tietze et al. 2013). There is more than one example of Neotropical linages that arrived from North America. Moreover, the arrival of Nearctic lineages to southern areas seems to be explained by the hypothesis of winter expansion ranges during the Miocene in Nearctic birds, which supports the northern origin of some Neotropical birds and posterior niche conservatism in the Neotropics (Kondo et al. 2008; Rolland et al. 2014; Winger et al. 2014; Zink and Gardner 2017). Furthermore, the factors that promoted the diversification of Euphoniinae in South America are still unclear because the study by Imfeld et al. (2020) did not represent the subregions in the Neotropics. This study aims to reconstruct the biogeographic history of Euphoniinae using a calibrated phylogeny and ancestral areas reconstruction based on current species ranges, South American Neotropical biomes, and Neotropical regionalization. Our main goal is to identify the biogeographic factors that led to the diversification of Euphoniinae, and our secondary objective is to reevaluate the phylogenetic relationships of Euphoniinae, using multiple samples of individual species and some allopatric subspecies to explore some intraspecific lineages for future research on the diversity of Euphoniinae. We also discuss the taxonomic implications of our findings.

## ﻿Materials and methods

### ﻿Sampling

We obtained 94 samples from the following collections: Louisiana State University Museum of Natural Science (LSU), The Field Museum of Natural History (**FMNH**), The Academy of Natural Sciences Philadelphia (**ANSP**), The Natural History Museum at the University of Kansas (**KU**), The Museo Alfonso L. Herrera Facultad de Ciencias (**MZFC**), The American Museum of Natural History (**AMNH**), and The Consejo Superior de Investigaciones Científicas (**CSIC**) (Suppl. material 1: table S1). This dataset included 23 tissue samples from the eight species of the genus *Chlorophonia* and 65 samples from 22 of 25 species of the genus *Euphonia*. Tissue samples were not available only for three species of Euphoniinae (*E.chalybea*, *E.trinitatis*, and *E.coccina*). We could not obtain DNA from toe pad samples, so they are not included in our analysis. We included samples from allopatric morphotypes of three subspecies: *Chlorophoniacyaneacyanea*, *C.musicasclateri*, and *C.musicamusica*. Also, we included representatives of the subspecies of three polytypic species: *E.chlorotica*, *E.violacea*, and *E.xanthogaster*. We included three representatives of the Carduelinae subfamily as outgroups: two samples of *Coccotharutesabeillei* and one of *Haemorhousmexicanus*. Samples deposited at the MZFC collection were obtained under a field collection permit provided by the Instituto Nacional de Ecología, SEMARNAT, Mexico (FAUT-0169). To estimate divergence times, we incorporated three samples of the Fringillinae subfamily: *F.coelebs*, *F.montifringilla*, and one sample of *Rhodinocichlarosea* representing the sister group to Fringillidae, the New World nine-primaried Oscines (See Divergence time estimation below).

### ﻿Laboratory processing and preparation for nextRAD sequencing

We extracted total genomic DNA from the tissue samples using the DNeasy tissues kit (Qiagen, Valencia, CA, USA) or the phenol: chloroform protocol (Hillis et al. 1996). The quality of DNA extractions was verified using gel electrophoresis. The DNA concentration was determined with a Qubit 3 fluorometer (ThermoFisher). The RAD sequence data was obtained using the nextRAD protocol (Russello et al. 2015) by the company SNPsaurus (http://snpsaurus.com/) (See Suppl. material 1: text S1 for details). The nextRAD libraries were sequenced on a single lane of an Illumina HiSeq 4000 with a single-end 150 bp protocol (University of Oregon). Raw sequence reads are available at GenBank SRA (BioProject accession PRJNA875486, see Suppl. material 1: table S1, the raw data are in the figshare repository: https://doi.org/10.6084/m9.figshare.20744656).

### ﻿Quality filtering and de novo alignment

We used IPYRAD 0.9.50 (Eaton and Overcast 2020) to filter the raw reads and perform de novo alignment of the nextRAD data. To filter reads by quality, we only retained reads with a Phred Q score of 43, and adapters were strictly filtered. We retained reads with more than 100 base pairs. We avoided variations due to sequencing errors by setting to 12 the minimum statistical depth and minimum depth for majority-rule base calling parameters. We set the maximum number of unique alleles to two and the maximum proportion of shared polymorphic sites per locus to 0.5. To minimize paralogues in the alignment we optimized the cluster threshold (CT) with five metrics. The first three of these metrics were proposed by McCartney-Melstad et al. (2019): (1) the correlation of pairwise divergence with pairwise missingness, (2) the mean bootstrap values in a maximum likelihood tree-building framework, and (3) the cumulative variance of the first three PCs. The fourth was the total number of loci and SNPs recovered (proposed by Mastretta-Yanes et al. 2015), and the fifth was the heterozygosity, proposed by Ilut et al. (2014). The rest of the parameters were set to their default values. The minimum number of samples with data for a locus to be included in the alignments was set to 43 (~ 50%). We performed the phylogenetic and time calibrated analysis using this final nextRAD sequence alignment.

### ﻿Laboratory processing and alignment for the ND2 marker

We amplified the mitochondrial marker ND2 (NADH Dehydrogenase Subunit 2; Sorenson 1999) via PCR in 12.5 mL reactions at a temperature of 54 °C. The sequencing was done by the Laboratorio de Secuenciación Genómica de la Biodiversidad y de la Salud, Instituto de Biología, UNAM. Sequence alignment was done with the algorithm MUSCLE (Edgar 2004) in the CIPRES Science Gateway (Miller et al. 2010). We included 68 sequences obtained from our tissue samples, for eight species of the genus *Chlorophonia* and 22 species of the genus *Euphonia* (GenBank accession numbers OP056102–OP056169, also see Suppl. material 1: table S1). Sequences were obtained from GenBank (Suppl. material 1: table S2) for *Chlorophoniaoccipitalis*, *E.affinis*, *E.godmani*, and *E.chlorotica*, including two samples from Imfeld et al. 2020 for *E.affinis* and *E.jamaica*. We also added outgroup species (one individual of *Haemorhousmexicanus*, one of *Coccothrautescoccothrautes*, and one of *Fringillacoelebs*) obtained from GenBank (Suppl. material 1: table S2).

### ﻿Phylogenetic analyses

For the alignment of the nextRAD sequences, we calculated the partitions and evolutionary models using PARTITIONFINDER 2 (Lanfear et al. 2016) under the following configuration: branch lengths linked, rcluster search algorithm (Lanfear et al. 2012), and Bayesian Information Criterion (BIC) for model selection. Then, a Maximum Likelihood (ML) tree was generated using RAXML v. 8.0.0 (Stamatakis 2014) in the CIPRES Science Gateway (Miller et al. 2010), using the partitions obtained above under a GTRGAMMA nucleotide substitution model with 1000 bootstrap replicates, using simple bootstrap analysis. For the mitochondrial ND2 marker, we obtained the evolutionary model for each codon position with PARTITIONFINDER 2 (Lanfear et al. 2012). Then, we generated a ML phylogeny with RAXML v. 8.0.0 (Stamatakis 2014), using the partitions obtained above under a GTRGAMMA nucleotide substitution model with 1000 bootstrap replicates.

### ﻿Time calibration tree

Divergence times were estimated for nextRAD sequences matrix with BEAST 2.6.3 (Bouckaert et al. 2019) implemented in the CIPRES Science Gateway (Miller et al. 2010). We assigned the partition schemes and evolutionary models obtained with PARTITIONFINDER 2 (Lanfear et al. 2012) (See section Phylogenetic analyses). We used secondary dating with a normal distribution and a calibration point based on the divergence between Fringillidae and the New World nine-primaried Oscines (17.1104 Mya; 95% HPD 14.7743–19.6278) calculated by Oliveros et al. (2019). For the molecular clock model, we selected the normal relaxed molecular clock following the recommendations of Drummond et al. (2006) and Li and Drummond (2012). We ran 50,000,000 generations, sampling every 1,000 generations, then we corroborated the effective sample size (ESS > 200) with TRACER v. 1.7.1 (Rambaut et al. 2018). Finally, we discarded the first 2,000 trees as burn-in and produced the maximum clade credibility tree with the highest 95% probability densities in TREE ANNOTATOR v. 1.8.0 (Rambaut and Drummond 2013). The tree was visualized with FIGTREE v. 1.4.1 (Rambaut and Drummond 2014).

### ﻿Biogeographic range estimation

We estimated the biogeographic history of Euphoniinae species with BioGeoBears (Biogeography with Bayesian and likelihood Evolutionary Analysis in R Scripts) (Matzke 2013). This package implements a likelihood version of Dispersal Vicariance Analysis (DIVA; Ronquist 1997), Dispersal-Extinction Cladogenesis (DEC) from the LAGRANGE program (Ree and Smith 2008), and BayArea and Bayesian Binary Model (BBM) (RASP; Yu et al. 2015). We did not include the +*j* due to controversy over these models (see Ree and Smith 2008; Ree and Sanmartín 2018; Matzke 2022).

We used the nextRAD matrix data to obtain a calibrated tree (see section above for specifications) and we collapsed the sampling tree to a specie tree as is suggested in the WikiSite of BioGeoBears (http://phylo.wikidot.com/biogeobears-mistakes-to-avoid#no_specimen_trees). Because we were unable to sample all subspecies across the subfamily, we performed the BioGeoBears analysis at the species level. We defined seven areas based on the current species distributions of Euphoniinae, the principal biomes of South America (Nores 2020) and the Biogeographical regionalization of the Neotropical region by Morrone (2014), using as reference the shapefiles by Löwenberg (2014): Caribbean; Mexican Transition Zone + Mesoamerica + Central America; Andes; Pacific W of Andes; Amazonas (the Amazon rainforest); Dry diagonal; and Paraná-Atlantic Forest. We allowed the occupation of up to three areas based on the ranges of the extant species. Finally, we compared the six different models for statistical fit via comparison of the Akaike weight (ωi) values. BioGeoBears (Matzke 2013) was used to obtain the range expansion (*d*), and range contraction (*e*).

## ﻿Results

### ﻿Quality filtering and de novo alignment

The percentage of reads that passed the quality filters was 97.66–98.95%, and the number of retained reads per sample ranged from 1,016,414 to 6,242,114. The optimal CT value was 0.87 (see the Suppl. material 1: Text S1 for details) Consensus sequence heterozygosis ranged from 0.00312 to 0.01760. The alignment used for phylogenetic inference recovered 2,570 loci and a total of 369,000 bp, the mean locus length was 141. The percentage of missing data for the sequence matrix was 37.43%, and the sample coverage ranged from 2165 loci to 311 loci (Suppl. material 1: tables S3, S4).

### ﻿Phylogenetic analyses

**Figure 1.**
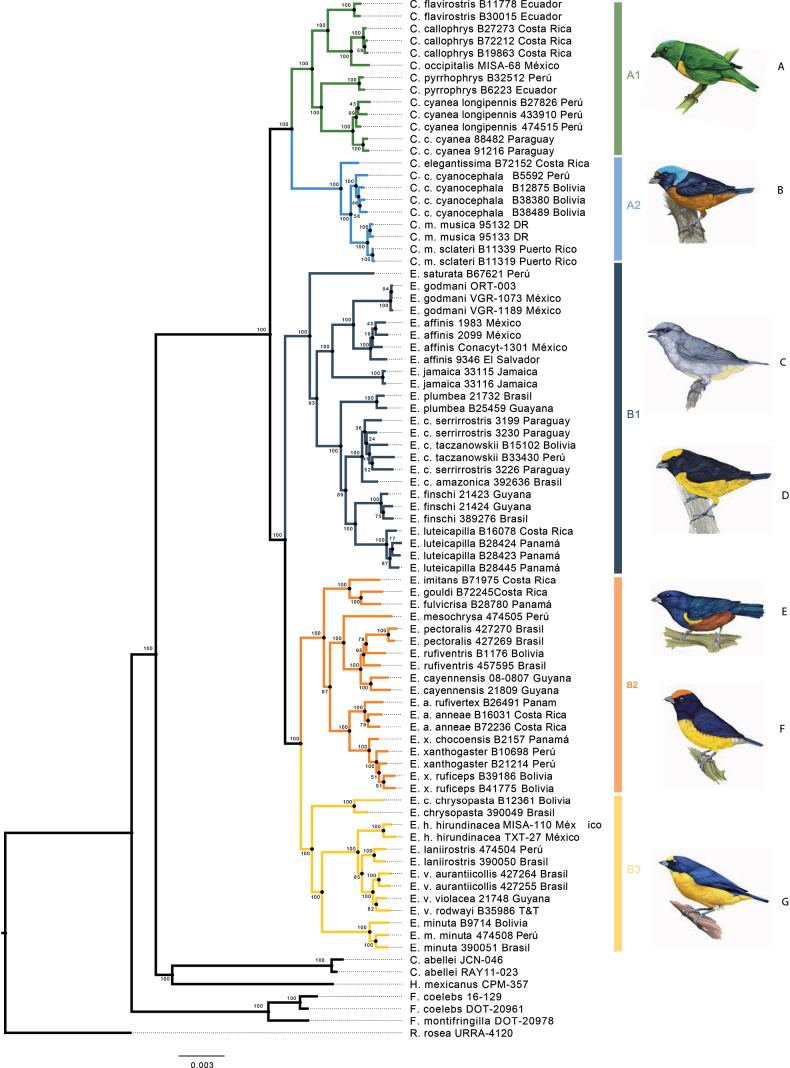
Maximum likelihood phylogeny with nextRAD data for Euphoniinae. A1, A2: genus *Chlorophonia*, B1, B2, and B3 genus *Euphonia*. From top to bottom, the illustrations depict **A***C.occipitalis***B***C.elegantissima***C***E.jamaica***D***E.luteicapilla***E***E.pectoralis***F***E.anneae***G***E.hirundinacea*. The illustrations were created by Germán García Lugo.

**Figure 2.**
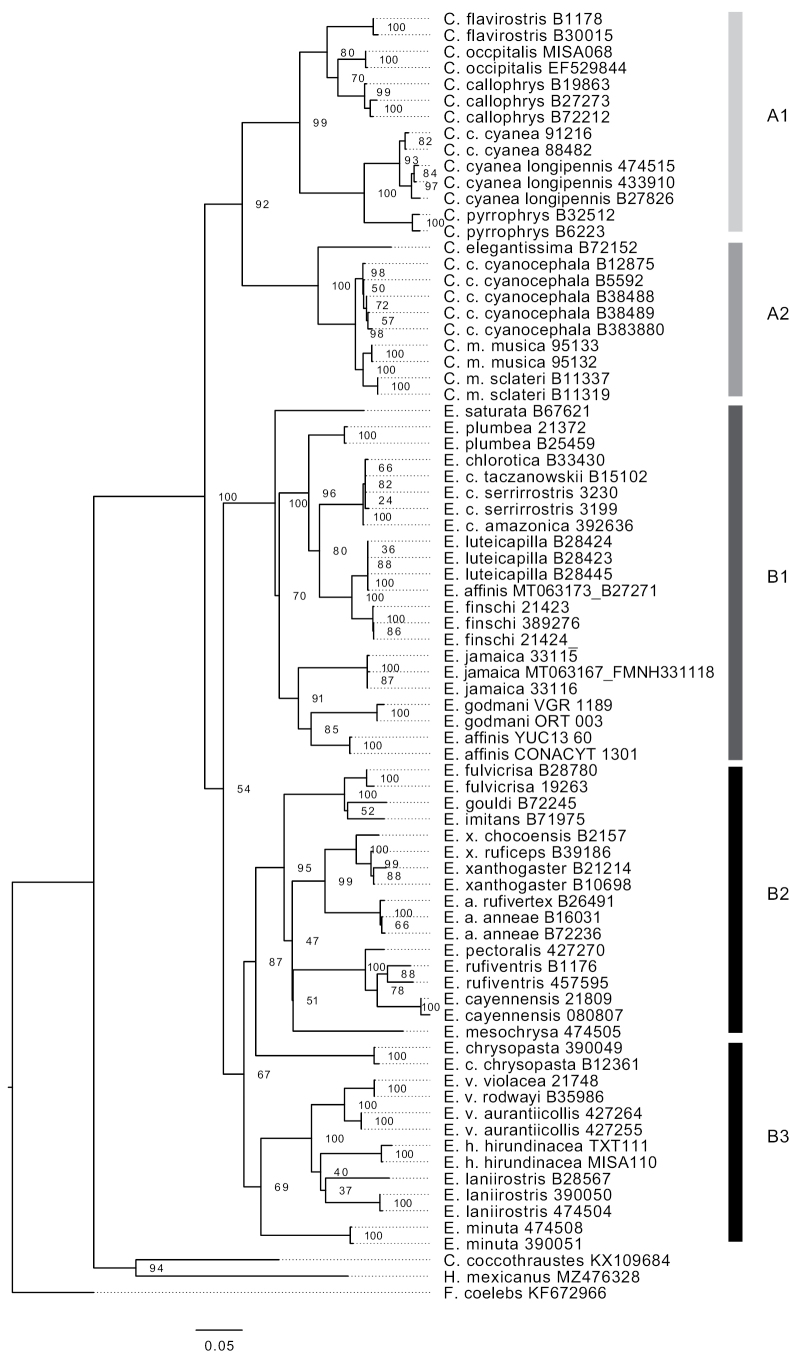
Maximum likelihood phylogeny based on ND2 data for Euphoniinae. A1 and A2: genus *Chlorophonia*, B1, B2, and B3 genus *Euphonia*.

For the nextRAD alignment, a total of 29 partitions were identified (See Suppl. material 1: table S5). The nextRAD ML phylogeny showed strong support for two main clades, A and B, which had two and three strongly supported subclades, respectively (A1, A2 and B1, B2, B3; bootstrap values for all five clades = 100) (Fig. 1). These clades were also recovered in the ND2 ML phylogeny with lower support (Fig. 2). Clade A1 contained the green *Chlorophonia* species: *C.callophrys*, *C.cyanea*, *C.flavirostris*, *C.occipitalis*, and *C.pyrrhophrys*. Clade A2 contained the Blue-headed Euphonias: *C.elegantissima*, *C.cyanocephala*, and *C.musica*. Clade B1 contained the blue-black throated Euphonias: *E.affinis*, *E.chlorotica*, *E.finschi*, *E.godmani*, *E.saturata*, *E.luteicapilla*, *E.plumbea*, *E.saturata*, and *E.jamaica*. The sample of *E.affinis* from the study of Imfeld et al. (2020) was in the *E.luteicapilla* clade. The B2 clade contained nine species that we refer as the rufous Euphonias since they have characteristic patches of rufous color on their bellies, crest and/or undertail-coverts: *E.anneae*, *E.cayennensis*, *E.fulvicrisa*, *E.imitans*, *E.gouldi*, *E.mesochrysa*, *E.pectoralis*, *E.rufiventris*, and *E.xanthogaster*). The B3 clade contained the yellow-throated Euphonias: *E.chrysopasta*, *E.hirundinacea*, *E.laniirostris*, *E.minuta*, and *E.violacea*).

The nextRAD phylogeny and ND2 phylogeny presented some differences in the clade B2 (Figs 1, 2). In the ND2, tree *E.gouldi* was closer to *E.imitans* (bootstrap value = 52), while in the nextRAD tree *E.gouldi* was the sister species of *E.fulvicrisa*. Interestingly, in the nextRAD phylogeny *E.rufiventris* was paraphyletic with respect to *E.pectoralis*, whereas *E.cayennensis* was strongly supported as monophyletic (bootstrap value = 100). However, in the ND2 phylogeny, *E.rufiventris* was closer to *E.cayennensis* (bootstrap value = 78), and *E.pectoralis* was the sister group (bootstrap value = 100). *E.chrysopasta* also showed disagreement in its phylogenetic relationships between the nextRAD and ND2 trees: in the nextRAD tree it was placed in clade B3 with the yellow throated-euphonias (bootstrap value = 100), while in the ND2 tree it was placed in the rufous Euphonias group (bootstrap value = 87) (Fig. 2).

Our sampling included allopatric subspecies with unique morphotypes for *C.musica* and *C.cyanea*. We found a split between *C.musicamusica* from the Dominican Republic and *C.musicasclateri* from Puerto Rico. A split also was recovered between the *C.cyaneacyanea* populations from Paraguay and the rest of the *C.cyanea* samples. We included in the genus *Euphonia* intraspecific samples for *E.chlorotica*, *E.xanthogaster* and *E.violacea* species. For the species *E.chlorotica*, we included six samples, which represented four subspecies—*E.C.serrirrostris* from Paraguay, *E.chloroticaamazonica* from Brazil, *Euphoniachloroticaamazonica* from Brasil and *E.C.taczanowskii* from Bolivia and Peru (Hilty 2020b)—which formed a monophyletic group without phylogenetic structure within the group. For *Euphoniaxanthogaster*, the sample from Panama, *E.xanthogasterchocoensis*, was the sister taxon of the rest of the *E.xanthogaster* samples, and the samples from Cochabamba Bolivia formed a clade and represented the subspecies *E.x.ruficeps*. In *E.violacea*, our phylogeny recovered two clades, one for *E.v.rodwayi* from Trinidad and Tobago and *E.violaceaviolacea* from Guyana, and another from East Brazil, which corresponded to *E.violaceaaurantiicollis*.

### ﻿Calibrated tree

**Figure 3.**
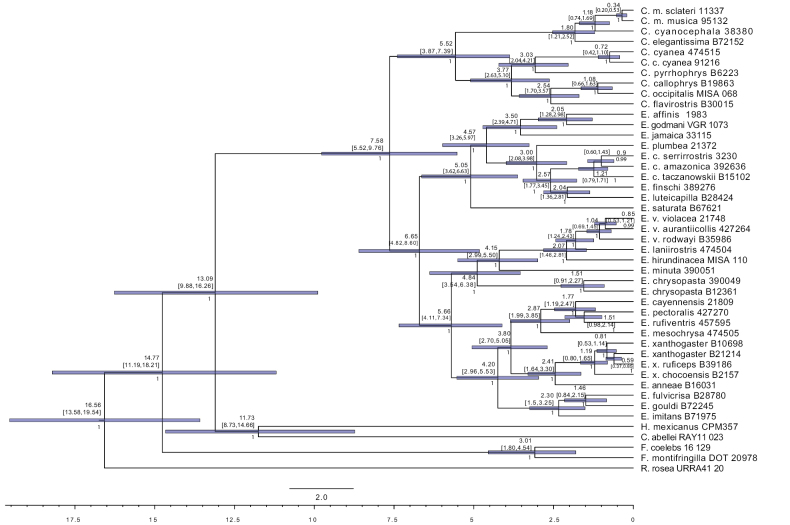
Time Calibrated Tree based on nextRAD data for Euphoniinae.

The Euphoniinae crown age was 7.58 Mya ago (95% HPD= 5.52–9.76) (Fig. 3). The *Chlorophonia* node split 5.52 Mya (95% HPD = 3.87–7.39), with the blue-headed *Chlorophonia* originating 1.8 Mya (95% HPD = 1.21–2.52) and the green *Chlorophonia* node emerging 3.77 Mya (95% HPD = 2.63–5.10) (Fig. 3). The *Euphonia* crown age was estimated at 6.65 Mya ago (95% HPD = 5.28–9.13). Within *Euphonia*, the clade B1 node originated 5.05 Mya (95% HPD = 4.06–7.12), and clade B2-B3 5.66 Mya (95% HPD = 4.58–7.77), B2 node 4.20 Mya (95% HPD = 2.96, 5.53), B3 4.15 Mya (95% HPD = 2.99, 5.50) (Fig. 3).

### ﻿Biogeographic range estimation

**Figure 4.**
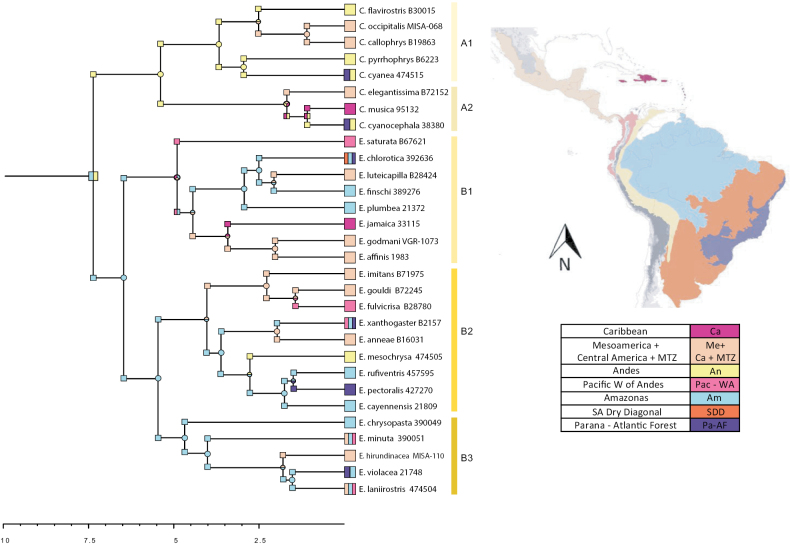
Biogeographical ancestral area reconstruction from BioGeoBEARS. Time Calibrated Tree with hypothetical ancestral areas and present areas and Biogeographical areas used in this study. The areas were mapped using ArcGIS (ArcMAP 10.2.2; Esri, Redlands, CA, USA) and the Biogeographic Regionalization on the Neotropical region shapefiles (Löwenberg 2014; Morrone 2014) see the text for more information.

Our BioGeoBEARS results suggested that Fig. 4 was the best supported biogeographic model for our data, with an Akaike weight (ωi) of 0.0004 (Table 1) (See Suppl. material 1: figs S1–S3). The range expansion *d* value was 0.041, and the value of range extinction was of 1.0e-12. According to our results, the Euphoniinae ancestor established in northern South America, Andes, and Amazonas 7.58 Mya ago. Then, the Euphoniinae ancestral populations split into two main clades, *Euphonia* in the lowlands of Amazonas and *Chlorophonia* in the Andes. The blue-headed *Chlorophonia* ancestor was widespread in Mesoamerica, Caribbean, and South America, and the green *Chlorophonia* ancestor remained in the Andes. Within the blue headed *Chlorophonia*, *C.elegantissima* split in Mesoamerica. In contrast, the ancestor of *C.cyanocephala* and *C.musica* split in the Andes and the Caribbean. Currently, *C.cyanocephala* occupies two regions—the Andes and the Paraná-Atlantic Forest (by *C.cyanocephalacyanocephala*). *Chlorophoniamusica* split in the Caribbean. Then, within the green *Chlorophonia*, *C.cyaneacyanea* invaded the Paraná-Atlantic Forest, and *Chlorophoniapyrrhophrys* stayed in the Andes. Finally, the ancestor of *Chlorophoniaflavirositrs*, *C.occipitalis*, and *C.callophrys* moved northward and colonized Mesoamerica by range expansion. The first split event of *Euphonia* occurred in the biogeographic area of Amazonas, following by the split of the three main clades of *Euphonia* with different ancestral area: 1. Amazonas, Pacific W Andes, and Mesoamerica for Euphonia B1, 2. Amazonas and Mesoamerica for B2, and 3. Amazonas for B3. In the three main clades, posterior speciation events involved range expansion to trans-Andes areas such as Mesoamerica (by *E.imitans*, *E.gouldi*, *E.fulvicrisa*, *E.anneae*, *E.minuta*, *E.hirundinacea*, *E.luteicapilla*, *E.affinis*, and *E.godmani*), and the Pacific (by *E.fulvicrisa*, *E.saturata*, and *E.xanthogaster*). There were also dispersals into other South American areas: Paraná-Atlantic Forest (by *E.xanthogaster*, *E.pectoralis*, *E.violacea*, and *E.chlorotica*) and the Andes area (by *E.mesochrysa*).

**Table 1.** BioGeoBEARS analysis models, parameters, and scores Models. Values of Log-Likelihood (LnL), Numbers of Parameters (P), *Range expansion* (*d*), *Range Contraction* (*e*), Akaike Information Criterion (AIC), and Akaike weight (ωi).

Model	LnL	P	*d*	*e*	AIC	ωi
DEC	-98.32	2	0.039	0.032	200.6	2.9e-06
DIVALIKE	-93.32	2	0.041	1.0e-12	190.6	0.0004
BAYAREALIKE	-103.2	2	0.043	0.26	210.5	2.1e-08

## ﻿Discussion

This study aimed to identify the biogeographic patterns of Euphoniinae in the Neotropical Region and enhance our understanding of their phylogenetic relationships. The analysis revealed that the Euphoniinae ancestor likely migrated to the Neotropics from North America and arrived to South America via the Isthmus of Panama. In the Neotropics, the establishment of the Western Amazonas and the Northern Andean Miocene pulse likely led to vicariance events between *Chlorophonia* and *Euphonia* ancestor. The ancestral range of *Chlorophonia* was in the Andes, whereas that of *Euphonia* was in the Amazonas. Speciation occurred in situ in the Andes for the green *Chlorophonia*, and the blue-headed *Chlorophonia* were widespread in the Neotropical highlands and the Caribbean. In *Euphonia*, some lineages diversified in trans-Andean areas by range expansion, while others reached South America’s eastern zones, including the eastern Amazonas, Dry Diagonal, and Atlantic Coast. The findings are mostly consistent with the phylogeny reported by Imfeld et al. (2020) and other morphologically based groupings (Sibley and Monroe 1990; Isler and Isler 1999). Based on the results, in the discussion we propose genus-level taxonomic rearrangements.

### ﻿Phylogenetic relationships

Our analysis of the phylogenetic relationships between Euphoniinae has identified two main groups, known as *Chlorophonia* and *Euphonia*, according to the current classification by the American Ornithological Society (Chesser et al. 2021). Within *Chlorophonia*, we have identified two distinct subgroups: *Chlorophonia* sensu stricto and the blue-headed *Chlorophonia*, subgroups A1 and A2 respectively, as shown in Figs 1, 2. These findings have been strongly supported by high bootstraps in both the nextRAD and ND2 phylogenies, which are consistent with the work of Imfeld et al. (2020). The relationships among the eight *Chlorophonia* species are also consistent with the findings of Imfeld et al. Our nextRAD phylogeny has confirmed that *Chlorophoniaoccipitalis* and *C.callophrys* are sister species, as previously shown in the mitochondrial phylogeny of Imfeld et al. (2020). Although some authors previously considered these to be a single species (Isler and Isler 1999), they are now recognized as separate species due to differences in their physical characteristics and geographic distributions.

The genus *Euphonia* is separated into three main groups (B1, B2, and B3) in both the nextRAD (Fig. 1) and the ND2 (Fig. 2) phylogenies. We will now present the significant findings for each of these groups in *Euphonia*. The *Euphonia* B1 group consists of the species from group 2 suggested by Isler and Isler (1999) plus *E.jamaica*, which was not associated with any species in Isler and Isler (1999). The coloration patterns support the evolutionary relationships between the members of this group, with the basal coloration pattern being the blue-black throat and yellow belly found in *E.saturata* and its two sister groups. One of these groups is the clade of *E.affinis* + *E.godmani*, whose sister group is *E.jamaica* (Figs 1, 2). Our calibrated phylogeny and ancestral reconstruction (Figs 3, 4) suggest that the ancestral lineage of these three species established in Mesoamerica and Central America from South America 2.39–4.71 Mya, likely due to the closure of the Panama Isthmus and the establishment of tropical forest in this area (Smith and Klicka 2010). Later, *E.affinis* and *E.godmani* split due to the formation of dry forest vegetation in the west Mesoamerica lowlands (Vázquez-López et al. 2020). The ancestral population of *E.jamaica* could have reached the island from Mesoamerica (see Fig. 4), and its unique color pattern is likely the result of environmental adaptations to the island, as suggested by Pregill and Olson (1981; cited in Isler and Isler 1999). In another group, we find *E.luteicapilla* as the sister taxon of *E.finschi*, and these two taxa formed a group with *E.chlorotica* and *E.plumbea*. Our phylogeny is consistent with the geographic distribution of this group, as these species are distributed from Panama to South America with allopatric distributions. The predominant color pattern is blue-black throat and yellow belly, though *E.plumbea* is an exception. The species *E.trinitatis* and *E.concinna* were not included in the present study but were placed in this group by Imfeld et al. (2020), which did not include *E.godmani*. In this work, *E.affinis* + *E.godmani* are the sister group of *E.jamaica*, rather than *E.luteicapilla*, as previously reported by Imfeld et al. (2020). Indeed, in the ND2 phylogeny, the *E.affinis* sample of Imfeld et al. (2020) (MT063173) is embedded in the *E.luteicapilla* group, suggesting that the *E.affinis* sample of Imfeld et al. (2020) may have been misidentified. Future phylogenetic research that samples these species with more than one specimen could provide new information about this group’s evolutionary relationships and diversification.

The phylogenetic relationships in the B2 and B3 clades are similar in those in the Imfeld et al. (2020) phylogeny, as well as the species groups of Sibley and Monroe (1990) and Isler and Isler (1999). Although these clades are sisters, they have differences in their color patterns, indicating species radiation. The *Euphoniaimitans*, *E.gouldi*, and *E.fulvicrisa* species are in one clade, while *E.anneae* and *E.xanthogaster* are in another, consistent with the phylogeny of Imfeld et al. (2020) and previous species groups. The phylogenetic relationships coincide with coloration and geographic distribution, as these species are allopatric. Another clade contains *E.mesochrysa*, *E.pectoralis*, *E.rufiventris*, and *E.cayennensis*, with high support for the nextRAD phylogeny. The phylogenetic relationships of *E.pectoralis*, *E.rufiventris*, and *E.cayennensis* are less clear due to discordance between nuclear and mitochondrial data. Despite the uncertain relationships, the phylogenetic patterns could result from the history of the distribution of their habitat. These species inhabit humid forests with allopatric distributions in the Amazonas and the Atlantic Forest. However, evidence suggests that these biomes may have been in contact in the past, through corridors across the Dry Diagonal or the Northern Areas of the Atlantic East Coast (Prates et al. 2016; Trujillo-Arias et al. 2018). Further research is needed to resolve these species’ phylogenetic relationships and biogeographic patterns. The B3 clade includes five species, with *E.violacea*, *E.laniirostris*, and *E.hirundinacea* forming a group due to their similar coloration (Isler and Isler 1999). Meanwhile, *E.minuta* and *E.chrysopasta* form their own group in Isler and Isler (1999). The ND2 phylogeny shows different relationships, with *E.chrysopasta* as the external branch of the B2 clade in the mitochondrial tree and as the external branch of the clade B3 in the nextRAD phylogeny. The relationship between *E.hirundinacea* and *E.laniirostris* may be inaccurate due to the lack of *E.chalybea* in the phylogeny, which is the external branch of these three species in the Imfeld et al. (2020) phylogeny. Further research is needed to resolve these species’ phylogenetic relationships and biogeographic patterns.

Our research discovered distinct lineages in both *C.cyanea* and *C.musica* allopatric morphotype samples, which were strongly supported in the nextRAD phylogeny. The *C.cyaneacyanea* in the Atlantic Forest exhibited a different coloration pattern compared to other subspecies (Hilty and Bonan 2020), and the calibrated phylogeny backed the split for the *C.cyaneacyanea* clade 0.72 Mya ago. This may have been due to movement from the East Andes to the Atlantic Forest, crossing the corridor in the dry areas of the Cerrado and Chaco (Trujillo-Arias et al. 2017). *Chlorophoniamusica* has two distinct clades in our phylogenies, one from Puerto Rico and one from the Dominican Republic. *Chlorophoniamusica* has three subspecies with allopatric distribution and differences in coloration, leading to these subspecies being considered separate species on multiple occasions (Greeney 2021). The phylogenies show intraspecific lineages for *E.xanthogaster* and *E.violacea* (Figs 1, 2). More extensive sampling is required to evaluate species boundaries in *E.xanthogaster* due to geographic variation and large genetic distance between samples and remarkable geographic variation (Hilty 2020a; Imfeld et al. 2020). Samples of *E.violacearodwayi* and *E.violaceaviolacea* formed a monophyletic group, while *E.violaceaaurantiicollis* formed its own clade; this subspecies is larger and isolated in the Atlantic Forest. These findings emphasize the importance of future studies with extensive sampling to better understand species limits and diversification in Euphoniinae (Vázquez-López et al. 2020).

### ﻿Neotropical biogeographic patterns in Euphoniinae

Our analysis using BioGeoBears indicated that the biogeographic model DIVALIKE is the most suitable (see Table 1). The models suggest that range expansion and vicariance were the primary mechanisms behind the diversification of Euphoniinae in the Neotropical Region (see Table 1). Euphoniinae was present in South America ~ 7.58 million years ago (5.52–9.76 Mya) (Fig. 4). This suggests that the Euphoniinae lineage could have reached South America from North America across the first emergences of the Isthmus of Panama (Morgan 2008; Verzi and Montalvo 2008; Bacon et al. 2015; Montes et al. 2015). It is possible that the Euphoniinae ancestor colonized the Neotropics from North America (Oliveros et al. 2019) due to the expansion of their winter ranges during the Miocene climate changes, followed by range contraction in the northern areas. There is substantial evidence of this pattern in Neotropical decedents of temperate avian lineages (Kondo et al. 2008; Rolland et al. 2014; Winger et al. 2014; Zink and Gardner 2017). After diversifying in South America, Euphoniinae could not recolonize temperate zones due to niche conservatism (Winger et al. 2019). Our results suggest that the two main lineages of Euphoniinae occupied the Andes and Amazonas during the late Miocene (Fig. 4), with *Chlorophonia* (5.52 Mya) in the Andes and *Euphonia* (6.65 Mya) in the Amazonas (Figs 3, 4). This pattern could result from the Central and North Andes uplift pulses and the establishment of the Western Amazonas (Antonelli et al. 2010; Hoorn et al. 2010; Carneiro et al. 2018). The *Chlorophonia* genus has an ancestor that was primarily found in the Andes mountains, and only a few subspecies (*C.cyanea*, *C.musica*, and *C.cyanochephala*) have dispersed to lowlands. Similar patterns have been observed in other Andean bird species (Sedano et al. 2010; Beckman and Witt 2015). The biogeographic results suggest that the majority of speciation events for the A1 clade occurred in the Andes, with some range expansion events to the Central American Forests and one event of dispersion to Eastern South America. Meanwhile, the ancestor of the A2 clade dispersed in Mesoamerica, the Andes and the Caribbean ~ 1.8 million years ago, before *C.elegantissima* split in Mesoamerica and after *C.musica* split in the Caribbean (Fig. 3); similar patterns were found by Imfeld et al. (2020). In contrast, the biogeographic results for the genus *Euphonia* suggests that the ancestor of the main clades of *Euphonia* originated in the Amazonas region, likely in the western area; this pattern has been previously observed in other bird taxa (Carneiro et al. 2018; Silva et al. 2019). Allopatric distributions are common among sister species in *Euphonia*, this could be resulted from range expansion and some dispersal events to new areas followed by vicariance, as the biogeographic results indicate. The three main clades of *Euphonia* have different ancestral areas, and there were two independent trans-Andean migration events: Pacific W Andes and Mesoamerica for *Euphonia* B1 and Mesoamerica for *Euphonia* B2. Meanwhile, B3 remained in the Amazonas. Further discussion on the areas and timing of Euphoniinae diversification in the Neotropics can be found below.

### ﻿Trans-Andean diversification and the role of the Isthmus of Panama

According to the biogeographic analysis, multiple invasions to trans-Andean areas occurred with different origins and ages in both *Euphonia* and *Chlorophonia*. During the Pliocene, the *Euphonia* B1 and B2 lineages moved from the Amazonas to trans-Andean areas, while another three lineages split after reaching the trans-Andean areas during the Pleistocene: *E.luteicapilla*; *E.anneae* – *E.xanthogaster*; and the ancestor of *E.hirundinacea*, *E.laniirostris*, and *E.violacea*. The Pliocene splits from the Amazon could be explained by the isolation of the lowland forests west of the Amazon basin by the final Northern uplift (8–4 Mya) (Gregory-Wodzicki 2000; Pérez-Consuegra et al. 2021), while the younger trans-Andean lineages could be explained by dispersal events after the northern Andean uplift. Additionally, the analysis suggests that *E.mesochrysa* split in the Andes, after the range expansion of its ancestor. For *Chlorophonia*, diversification in the trans-Andean areas involved range expansion to Central America from the Andes. The A2 clade was in northern areas, and *C.elegantissima* split in Mesoamerica. This pattern agrees with the previous report for Euphoniinae (Imfeld et al. 2020) and with the sinking finches (Beckman and Witt 2015). Overall, the trans-Andean species established in Mesoamerica and Central America during the Pliocene and Pleistocene, which could be the effect of the entirely closed of the Isthmus of Panama and Neotropical habitats outside of South America, before 3.5 Mya ago (Smith and Klicka 2010; Bacon et al. 2015; Imfeld et al. 2020).

### ﻿Diversification in the Cis Andean areas

The distribution patterns of Euphoniinae in the East side of South America contrast between *Chlorophonia* and *Euphonia* since the analyses suggest that *Chlorophonia* reached east areas from the Andes and *Euphonia* reached east areas from the Amazonas (Fig. 4). The Atlantic Forest is separated from the East South Andean Forest by the dry diagonal of South America. However, many related avian lineages inhabit both biomes, which suggests a past connection. Additionally, paleoclimatic studies suggest that the East South Andean Forest and Atlantic Forest were connected during the Quaternary by cyclical corridors of humid forest in the currently dry areas of the Cerrado and Chaco (Trujillo-Arias et al. 2017). This evidence, along with our phylogenetic analysis, suggest that *C.cyanea* could have reached the Atlantic Forest by the Cerrado and Chaco corridors ~ 0.7233 Mya (Fig. 3), consistent with other endemic lineages of the Atlantic Forest (Trujillo-Arias et al. 2017, 2018; Cabanne et al. 2019). In *Euphonia*, older taxa are found in the western area of the Amazonas and younger taxa in the areas east of the Amazon basin (Fig. 4): *E.plumbea*, *E.finschi*, and *E.chlorotica*. This pattern could be the result of a combination of factors such as Andean uplift, Pleistocene climate, and changes in the river (Hoorn et al. 2010; Carneiro et al. 2018; Silva et al. 2019). However, a detailed description of the Amazonian diversification of these taxa is outside the scope of this work. The Amazonas is isolated from the Atlantic Humid Forest by the Dry Diagonal, and some *Euphonia* species have allopatric populations in the Atlantic Forest. *Euphoniachlorotica* is an exception, as it migrated from the Amazon River to the Dry Diagonal and the Atlantic Forest ~ 2.57 Mya, probably during the Plio-Pleistocene expansion of the Dry Diagonal (Coscaron and Morrone 1998; Luebert et al. 2011; Luebert 2021). Meanwhile, the related species, *E.rufiventris*, *E.cayennensis*, and *E.pectoralis*, have separate distributions in the Western-Central Amazonas, East Amazonas, and Atlantic Forest, respectively. According to the calibrated tree and the biogeographic analysis, it is likely that *E.pectoralis* arrived in the Atlantic Forest from either the Southeast Amazonas or Northeastern Brazil, the corridors proposed between the Amazonas and Atlantic Forest (Prates et al. 2016; Trujillo-Arias et al. 2018). Similar phylogenetic patterns were found for *E.violaceaaurantiicollis*, an Atlantic Forest subspecies. Other *Euphonia* species also inhabit the Atlantic Forest area, such as *E.xanthogasterxanthogaster*, *E.chalybea*, and *E.chloroticaamazonica*, but were not included in the biogeographic analysis. Further research on phylogeography will help us better understand the biogeographic pattern for each *Chlorophonia* and *Euphonia* species in the East Atlantic Coast area.

### ﻿Caribbean invasions

In Euphoniinae, two species are distributed in the Caribbean: *E.jamaica* and *C.musica*. The biogeographic analysis conducted has revealed that they migrated to the Caribbean from continental North American and South American regions at different times. The findings suggest that the ancestor of *C.cyanocephala* – *C.musica* was in South America and the Caribbean during the Pleistocene period, which occurred ~ 1.18 million years ago, possibly traveling in a northward direction from the Andes as was described by Imfeld et al. (2020). Meanwhile, the *E.jamaica* lineage arrived in the Caribbean earlier, ~ 3.5 million years ago, after migrating from the Mesoamerica-MTZ-CA area, possibly over water. The Parsimony Biogeographic Patterns of the Caribbean Basin have revealed that the Greater Antilles and Yucatan-Central American countries are sister areas, nested from north to south (Vázquez-Miranda et al. 2007).

### ﻿Taxonomic revisions

Imfeld et al. 2020 proposed the resurrection of *Cyanophonia* (Bonaparte, 1851) as the genus of blue-headed *Euphonia* and they assigned *Cyanophoniamusica* as the type species for the genus (Gmelin 1789). Type locality: Santo Domingo, Dominican Republic). However, in the Sixty-second Supplement to the American Ornithological Society’s Checklist of North American Birds (Chesser et al. 2021), the three blue-headed *Euphonia* were merged into the genus *Chlorophonia*. We believe that the blue-headed Euphonia taxonomy needs to be reconsidered, and we also recommended the resurrection of *Cyanophonia* (Bonaparte, 1851) to denote the three species of blue-headed *Chlorophonia*. Furthermore, three lines of evidence suggest an independent evolutionary history of these three species from the rest of *Chlorophonia*:

1. The differences in the color pattern (Fig. 1) — the blue head patches are a shared character between the *Chlorophoniasensu stricto* and blue-headed *Chlorophonia*. However, *Chlorophoniasensu stricto* has a predominantly green coloration, while blue-headed *Chlorophonia* have a rufous belly and glossy dark blue back and throat.
2. The phylogenetic distinction — all the phylogenies so far show a well-supported split into two clades.
3. The biogeographic patterns also show differences between these groups — both share an ancestral population in the Andes, but in *Chlorophoniasensu stricto* cladogenesis continued in the Andes and from there reached other South American Areas and Mesoamerica; meanwhile, the blue-headed *Chlorophonia* lineages were widespread in North and South America. Imfeld et al. (2020) also reported these differences in the biogeographic patterns.

We also propose that the *Euphonia* taxonomy be reviewed, since this is a larger group than *Chlorophonia* – *Cyanophonia*, with three phylogenetic groups that also display morphological particularities.

1. The first group include the groups proposed by Isler and Isler (1999), 1 *Euphoniajamaica* and 2 – *E.saturata*, *E.chlorotica*, *E.luteicapilla*, *E.finschi*, *E.plumbea*, *E.godmani*, *E.affinis*, *E.trinitatis*, and *E.concinna*. These species inhabit lowlands habitats and display the classic *Euphonia* pattern of dark blue throat with yellow belly (Fig. 1). Even *E.plumbea* displays similar patterns but with paler coloration. *Euphoniajamaica* has a classic finch bill and does not have the pattern of a black throat; however, we assign it to this group because of its phylogenetic position.
2. The second group include the Isler and Isler (1999) group 3: *E.violacea*, *E.laniirostris*, *E.hirundinacea*, *E.chalybea*; group 6 *E.minuta*; and *E.chrysopasta* from group 7. These species display a pattern of yellow throat, yellow crest, and black back that can have purple, blue or green gloss. In this group *E.chrysopasta* and *E.minuta* do not show yellow throat plumage, however, the females display a similar pattern to *E.hirundinacea* and *E.chalybea*.
3. The third group has nine species of three of the groups Isler and Isler (1999); group 5: *E.fulvicrissa*, *E.imitans*, *E.gouldi*, *E.mesochrysa*; group 7: *E.anneae*, *E.xanthogaster*; and group 8: *E.rufiventris*, *E.pectoralis*, and *E.cayennensis*. The character that defines this group is the rufous color patches, which can be on the belly, the crest and/or the undertail-coverts, in adult males and females.

The genus *Euphonia* was established by Desmarest (1806) with a female specimen of *Euphoniaolivacea*, which is listed as a synonym of *Euphoniaminuta* in the ‘Histoire naturelle des Tangaras, des Manakins et des Todiers’ by name (date), but without a clear description for the genus:

*The bird for which we give a figure named Euphone olive is entirely in a different case. It does not have very bright colors, and its small size makes us suspect that it is a female or a young individual, but we do not know to what species to refer it because its plumage presents no clue which could serve to establish a connection. It was recently sent to the Jardin des Plantes among many birds from Cayenne*.

Bonaparte (1851) suggested the genus *Pyrrhuphonia* for finch-billed *Euphonia*, the type species of which is *Euphoniajamaica* (Linnaeus, C 1766) (type locality: Jamaica). Cabanis (1861) suggest the genus *Phonasca* for *Euphonia* similar to *E.chlorotica* and *E.violacea*, and he listed the following species as *Phonasca*: *E.chlorotica*, *E.xanthogaster*, *E.fulvicrissa*, *E.trinitatis*, *E.luteicapilla*, *E.affinis*, *E.minuta*, *E.chalybea*, *E.hirundinacea*, *E.laniirostris*, and *E.violacea*. In that work, he described *E.saturata* and *E.luteicapilla*. Many *Euphonia* species were described under the genus *Tanagra* (Linnaeus 1764 or 1766); however, this genus is unavailable because it has been suppressed and placed on the Official Indices of Rejected and Invalid Names in Zoology (ICZN Opinion 852, Bull. Zool. Nom., 25: 74–75, 27 September 1968), since it was described twice for very different species. Also, this genus was widely used to name species that now are in different families (e.g., Thraupidae: *Anisoganthusnotabilis*, Passerillidae: *Chlorospingusflavopectus*, Cardinalidae: *Pirangaludoviciana*, Fringillidae: *Euphoniaaffinis*).

Consequently, we propose that the blue-black throated group remain as *Euphonia* (Desmarest, 1806), since it is the core group of the “true” Euphonia clade, with ten species, and these species display the characteristic pattern coloration of euphonias. The oldest *Euphonia* species in this group were described by Linnaeus (1766)—*E.jamaica* and *E.chlorotica*—of these two only *Euphoniachlorotica* has the classic euphonia pattern coloration. The yellow-throated group could be named *Phonasca* (Cabanis, 1861), which was previously nominated for five of the six species of this group by Cabanis (1861). We designate the Violaceus Euphonia, *Phonascaviolacea* as the type species, since it is the first species described in 1758 by Linnaeus. Finally, we propose a new genus for the rufous clade: *Rufiphonia* gen. nov. based on their rufous patches, and we designate the Rufous-bellied *EuphoniaRufiphoniarufiventris* (Vieillot, 1819) as the type species. We propose a new genus because there is no available name for a third group in *Euphonia*.

#### ﻿ Rufiphonia

Taxon classificationAnimaliaPasseriformesFringillidae

Vázquez-López & Hernández-Baños gen. nov.

2C2D9176-4AAB-596A-8361-C4635F678C4C

https://zoobank.org/D112E1E4-502C-438F-BF66-2C081D6D5745

##### Type species.

*Rufiphoniarufiventris* (Vieillot, 1819). Type locality: Perú.

##### Included species.

*Rufiphoniafulvicrissa* (Sclater, 1857), type locality: Santa Martha, New Granada; *R.imitans* (Hellmayr, 1936), type locality: El Pozo, Rio Terraba, Costa Rica; *R.gouldi* (Sclater, 1857), type locality: Guatemala; *R.mesochrysa* (Salvadori, 1873) type locality: No locality given, Bogotá, Colombia; *R.anneae* (Cassin, 1865), type locality: Santa Rosa, Costa Rica, *R.xanthogaster* (Sundevall, 1834), type locality: Río de Janeiro, Brazil; *R.pectoralis* (Latham, 1801), and *R.cayennensis* (Gmelin, 1789), type locality: Guyana.

##### Morphological description.

Most males of this genus display the classic *Euphonia* pattern of dark blue throat and back with yellow belly, with four exceptions. *R.gouldi* and *R.mesochrysa* have olive upper parts with grey-blue glosses. The males of *R.cayennensis* and *R.pectoralis* have predominantly dark and glossy metallic-blue plumage. Also, the males could have a forehead in yellow or rufous, a rufous belly, and undertail coverts in rufous. The females are primarily olive with contrasting rufous patches on the forehead, belly, or undertail coverts.

##### Diagnosis.

The new genus can be distinguished from all other *Euphonia* species by the rufous color patches, which can be on the belly, the crest, and/or the undertail-coverts, in both male and female adults.

## ﻿Conclusions

1. The nextRAD and ND2 phylogenies obtained in this study are generally consistent with the UCE and mitochondrial phylogenies of Imfeld et al. (2020).
2. Minor clades contained morphologically similar species with allopatric distribution ranges.
3. The biogeographic results suggest that the Andean uplift and the establishment of the western Amazonas drove the vicariance of *Chlorophonia* and *Euphonia* during the Miocene, with the Andes and the Amazonas as each ancestral area, respectively. The green *Chlorophonia* has an ancestor that was primarily found in the Andes mountains; after the establishment of the Isthmus of Panama, the *Chlorophonia* lineage reached the Central American and Mesoamerican highlands from the Andes. In contrast, *Euphonia* suggests that the ancestor of the main clades of *Euphonia* could have originated in the western Amazonas. The genus *Euphonia* reached trans-Andean areas from the Amazonas during the Pliocene and Pleistocene as a consequence of the vicariance of the west lowlands and the Amazonas. *Chlorophonia* and *Euphonia* species reached the Atlantic Forest biome during the Pleistocene, probably through the corridors that connected the East Andean Humid Forest and the Amazonas. *Chlorophonia* and *Euphonia* each had a Caribbean invasion with different geographic origins and ages.
4. We recommend recognizing the genus *Cyanophonia* for the species *Chlorophoniamusica*, *Chlorophoniacyanocephala*, and *Chlorophoniaelegantissima* since they represent a differentiated lineage in phylogeny, in their coloration patterns, and in their biogeographic history. Lastly, we propose a revision of the taxonomy of the genus *Euphonia* because there are three differentiated lineages at the phylogenetic, biogeographic, and morphological levels.

## Supplementary Material

XML Treatment for Rufiphonia

## ﻿Additional information

### Author ORCIDs

Melisa Vázquez-López https://orcid.org/0000-0002-1365-1860

Sandra M. Ramírez-Barrera https://orcid.org/0009-0005-3999-1934

Alondra K. Terrones-Ramírez hhttps://orcid.org/0000-0002-1486-0023

Sahid M. Robles-Bello https://orcid.org/0000-0003-1126-0150

Adrián Nieto-Montes de Oca https://orcid.org/0000-0002-8150-8361

Kristen Ruegg https://orcid.org/0000-0001-5579-941X

Blanca E. Hernández-Baños https://orcid.org/0000-0002-6222-4187

### Data availability

All of the data that support the findings of this study are available in the main text or Supplementary Information.

## Supplementary materials

Supplementary material 1 Supplementary information Data type pdf Explanation note **table S1.** List of specimens studied and their provenance. **table S2.** ND2 sequences obtained from Genbank. **table S3.** Results after filtering and clustering nextRAD data from Euphoniinae samples and outgroup samples. **table S4.** Sample coverage for nextRAD data from Euphoniinae samples and outgroup samples. **table S5.** Substitution models for nucleotide data partitions selected using the BIC in PartitionFinder. **text S1.** Laboratory process and sequence preparation for nextRAD sequencing. **text S2.** Quality filtering and Denovo alignment results. **figure S1.** DEC_maxareas3_v1 B=Caribbean C=Mesoamerica D=Andes E=Pacific F=Amazonas G=Chacoan H=Paraná-Atlantic Forest. **figure S2.** DIVALIKE_maxareas3_v1 B=Caribbean C=Mesoamerica D=Andes E=Pacific F=Amazonas G=Chacoan H=Paraná-Atlantic Forest. **figure S3.** BAYAREALIKE_maxareas3_v1 B=Caribbean C=Mesoamerica D=Andes E=Pacific F=Amazonas G=Chacoan H=Paraná-Atlantic Forest. https://binary.pensoft.net/file/963771This dataset is made available under the Open Database License (http://opendatacommons.org/licenses/odbl/1.0/). The Open Database License (ODbL) is a license agreement intended to allow users to freely share, modify, and use this Dataset while maintaining this same freedom for others, provided that the original source and author(s) are credited.Melisa Vázquez-López, Sandra M. Ramírez-Barrera, Alondra K. Terrones-Ramírez, Sahid M. Robles-Bello, Adrián Nieto-Montes de Oca, Kristen Ruegg, Blanca E. Hernández-Baños
