# Supplementary material for: ﻿Biogeographic factors contributing to the diversification of Euphoniinae (Aves, Passeriformes, Fringillidae): a phylogenetic and ancestral areas analysis
Source: Zookeys. 2024 Jan 8;1188:169–95. doi: 10.3897/zookeys.1188.107047 (PMC10790576; doi:10.3897/zookeys.1188.107047)
Supplement: Supplementary material 1 — Supplementary information [file zookeys-1188-169_article-107047__-s001.pdf]

Supplemental material.

- Table S1. List of specimens studied and their provenance.
- Table S2. ND2 sequences obtained from Genbank.
- Table S3. Results after filtering and clustering nextRAD data from Euphoniinae samples and outgroup samples.
- Table S4. Sample coverage for nextRAD data from Euphoniinae samples and outgroup samples.
- Table S5. Substitution models for nucleotide data partitions selected using the BIC in PartitionFinder.
- Text S1. Laboratory process and sequence preparation for nextRAD sequencing.
- Text S2. Quality filtering and Denovo alignment results.
- Figure S1. DEC_maxareas3_v1 B=Caribbean C=Mesoamerica D=Andes E=Pacific F=Amazonas G=Chacoan H=Paraná-Atlantic Forest.
- Figure S2. DIVALIKE_maxareas3_v1 B=Caribbean C=Mesoamerica D=Andes E=Pacific F=Amazonas G=Chacoan H=Paraná-Atlantic Forest.
- Figure S3. BAYAREALIKE_maxareas3_v1 B=Caribbean C=Mesoamerica D=Andes E=Pacific F=Amazonas G=Chacoan H=Paraná-Atlantic Forest.

Table S1. List of specimens studied and their provenance.

Specie	ID sample	Genbank num. ND2	Genbank num. nextRAD	Collection	Locality	Latitude	Longitude
Chlorophonia callophrys	B19863	OP056114	SRR21430399	LSUMZ	3km ENE Villa Mills, Km 95 Villa Mills. Cartago ProvincEuphonia Costa Rica.	9.5817463	-83.692065
Chlorophonia callophrys	B27273	OP056116	SRR21430385	LSUMZ	1km N Villa Mills; Cerro Abarca. Cartago ProvincEuphonia Costa Rica.	9.5662622	-83.706539
Chlorophonia callophrys	B72212	OP056115	SRR21430374	LSUMZ	Navarro, Muneco. Cartago ProvincEuphonia Costa Rica.	9.8	-82.074167
Chlorophonia cyanea cyanea	91216	OP056122	SRR21430446	KU	San Rafael National Park. Caazaopa/Itapua. Paraguay.	-26.35	-55.5167
Chlorophonia cyanea cyanea	88442	OP056121	SRR21430447	KU	San Rafael National Park. Caazapa. Paraguay.	-26.35	-55.5167
Chlorophonia cyanea longipennis	B27826	OP056123	SRR21430384	LSUMZ	77km WNW Contamana. Loreto Deparment. Peru.	-6.91666667	-74.35
Chlorophonia cyanea longipennis	433910	OP056124	SRR21430350	FMNH	Paucartambo: Consuelo, 15.9 km SW Pilcopata. Cusco. Peru.	-13.0167	-71.4833
Chlorophonia cyanea longipennis	474515	OP056125	SRR21430340	FMNH	Quebrada Huacabamba. Amazonas. Peru.	-6.5917	-77.5533
Chlorophonia flavirostris	B11778	OP056117	SRR21430391	LSUMZ	El Placer CA 670M. Esmeralda. Ecuador.	0.908611	-78.6167
Chlorophonia flavirostris	B30015	OP056118	SRR21430365	LSUMZ	Ca 2.7km E Alto Tambo. Esmeralda. Ecuador.	0.89638889	-77.465278
Chlorophonia occipitalis	MISA-68	OP056113	SRR21430354	MZFC	Villa Nueva, a 600 m por la carretera a Manuel Gutiérrez Nájera. Veracruz. Mexico.	19.7758889	-96.867722

Chlorophonia pyrrhophrys	B32512	OP056119	SRR21430364	LSUMZ	Quebrada Lanchal, ca (km ESE SalliquEuphonia Cajamarca Deparment. Peru.	-4.31666667	-78.75
Chlorophonia pyrrhophrys	B6223	OP056120	SRR21430378	LSUMZ	W slope Cordillera del Cutucú, S trail from Logroño to Yaupi. Morona-Santiago. Ecuador.	-2.72	-78.08
Chlorophonia cyanocephala cyanocephala	B12875	OP056105	SRR21430389	LSUMZ	Al W de la Ribera del Río Paucerna, 4 km río arriba del Río Iténez. Santa Cruz Deparment. Bolivia.	-12.891211	-63.045491
Chlorophonia cyanocephala cyanocephala	B38380	OP056104	SRR21430382	LSUMZ	Chuchial, ca 37 km SE Samaipata. Santa Cruz Deparment. Bolivia.	-17.6333333	-62.383333
Chlorophonia cyanocephala cyanocephala	B38388	OP056108	XXX	LSUMZ	Chuchial, approximately 37 km SE Samaipata. Santa Cruz Department. Bolivia	-18.3816	-63.62
Chlorophonia cyanocephala cyanocephala	B38489	OP056106	SRR21430381	LSUMZ	Chuchial, ca 37km SE Samaipata. Santa Cruz Deparment. Bolivia.	-17.6333333	-62.383333
Chlorophonia cyanocephala cyanocephala	B5592	OP056107	SRR21430379	LSUMZ	15 km by trail NE Jirillo on trial to Balsapuerto. San Martin Deparment. Peru.	-6.05	-76.733333
Chlorophonia elegantissima vincens	B72152	OP056103	SRR21430375	LSUMZ	Taalamanca, Tuba Creek. Limon. Costa Rica.	9.73472222	-81.104722
Chlorophonia musica musica	95132/8067	OP056111	SRR21430445	KU	Parque Nacional Sierra Baoruco, Pueblo Viejo. Santo Domingo. Dominican Republic.	18.2	-71.533333
Chlorophonia musica musica	95133	OP056112	SRR21430444	KU	Parque Nacional Sierra Baoruco, Pueblo Viejo. Santo Domingo. Dominican Republic.	18.2	-71.533333
Chlorophonia musica sclateri	B11319	OP056109	SRR21430400	LSUMZ	Llanos Costa; 0.7 km E mouth Arroyo Cazul. Cabo Rojo. Puerto Rico.	17.9757353	-67.133043

Chlorophonia musica sclateri	B11337	OP056110	SRR21430393	LSUMZ	Boqueron; Peñones de Melones, 1 km WNW intersection of routes 301 and 303. Cabo Rojo. Puerto Rico.	17.9966128	-67.183026
Euphonia affinis	1983	MT452146	SRR21430454	KU	24 km to S of SilvituChlorophonia CampechEuphonia Mexico.	18.2333	-90.2
Euphonia affinis	2099	MT452148	SRR21430453	KU	24 km to S of SilvituChlorophonia CampechEuphonia Mexico.	18.2333	-90.2
Euphonia affinis	9346	MT452149	SRR21430433	KU	Ánimas. Zacatecoluca. La Paz. El Salvador.	13.766357	-88.96689
Euphonia affinis	Conacyt-1301	MT452151	SRR21430359	MZFC	Nizanda Road to Sabanas. Asunción IxtaltepeChlorophonia Oaxaca. Mexico.	16.677953	-95.02317
Euphonia godmani	ORT-003	MT452159	SRR21430352	MZFC	3.5 km to Singayta. San Blas. Nayarit. Mexico.	21.6031	-105.2377
Euphonia godmani	VGR-1073	MT452167	SRR21430347	MZFC	Bajosori 10 km SO of Choix, Ejido 1. Municipio Choix. Sinaloa. Mexico.	26.65	-108.9
Euphonia godmani	VGR-1189	MT452164	SRR21430346	MZFC	Bajosori 10 km SO of Choix, Ejido 1. Municipio Choix. Sinaloa. Mexico.	26.65	-108.9
Euphonia anneae anneae	B16031	OP056164	SRR21430387	LSUMZ	4 km SE Virgen del Socorro. Heredia ProvincEuphonia Costa Rica.	10.2796644	-84.166342
Euphonia anneae rufivertex	B26491	OP056163	SRR21430394	LSUMZ	Dist. Gualaca, Cordillera Central, 4.3 km by road S lago Fortuna dam. Chiriquí Province Panama.	8.6974692	-82.229166
Euphonia anneae anneae	B72236	OP056165	SRR21430372	LSUMZ	Perez, Zeledon, Turrialba, Tausino. Cartago ProvincEuphonia Costa Rica.	9.78222222	-82.273333
Euphonia cayennensis	08_807	OP056153	SRR21430339	CSIC	Nouragues Natural Reserve, Regina, PararEuphonia Guayana.	4.033	-52.675
Euphonia cayennensis	21809/187876	OP056154	SRR21430451	ANSP	Iwokrmama Reserve; Essequibo River, 3 mi NW Kurupukari. Potaro-Siparuni. Guayana.	4.68333333	-58.7
Euphonia chlorotica serrirrostris	3230	MT452170	SRR21430434	KU	Bahia Negra, 14 km W, Estancia Triunfo. Alto Paraguay. Paraguay.	-20.0983333	-58.266667

Euphonia chlorotica amazonica	392636	OP056102	SRR21430438	FMNH	Monte Alegre, Campo do Desterro. Para. Brasil.	-18.8887294	-54.052851
Euphonia chlorotica serrirrostris	90102/3199	OP056133	SRR21430442	KU	Bahia Negra, 14 km W, Estancia Triunfo. Alto Paraguay. Paraguay.	-20.0983333	-58.266667
Euphonia chlorotica taczanowskii	B15102	OP056135	SRR21430388	LSUMZ	Velasco; 13km SW Piso firmEuphonia Santa Cruz Deparment. Bolivia.	-13.6900228	-61.67406
Euphonia chlorotica taczanowskii	B33430	OP056134	SRR21430363	LSUMZ	Las Juntas, junction of Rios Tabacomas and ChinchipEuphonia Cajamarca Deparment. Peru.	5.38333333	-77.233333
Euphonia chrysopasta	390049	OP056126	SRR21430441	FMNH	Cachoeira Nazare, W bank Rio Jiparana. Rondônia. Brasil.	-9.75	-61.916667
Euphonia chrysopasta chrysopasta	B12361	OP056127	SRR21430390	LSUMZ	Velasco 32km E Aserradero Moira, Preparque Noel kempff Mercado. Santa Cruz Deparment. Bolivia.	-14.3716801	-60.841165
Euphonia finschi	389276	OP056141	SRR21430443	FMNH	Fazenda Santa Cecilia, E bank Rio Branco, opposite Boa Vista. Roraima. Brasil.	2.7966332	-60.62297
Euphonia finschi	21423/187874	OP056139	SRR21430432	ANSP	Surama, bordering the Iwokrama ReservEuphonia Upper Takutu-Upper Essequibo. Guayana.	4.11666667	-59.05
Euphonia finschi	21424/187875	OP056140	SRR21430431	ANSP	Surama, bordering the Iwokrama ReservEuphonia Upper Takutu-Upper Essequibo. Guayana.	4.11666667	-59.05
Euphonia fulvicrisa fulvicrisa	B28780	OP056162	SRR21430366	LSUMZ	Achiote Road, Ca 2km-bridge at Rio Providencia. Colon Province Panama.	9.2267615	-80.030214
Euphonia fulvicrisa purpurascens	19263	OP056161	XXX	ANSP	20 Km ENE MuisnEuphonia Cabeceras de Bilsa. Esmeraldas. Ecuador.	-	-
Euphonia gouldi praetermissa	B72245	OP056159	SRR21430371	LSUMZ	Taalamanca, Tuba Creek. Limon. Costa Rica.	9.73472222	-81.113611

Euphonia hirundinacea hirundinacea	MISA-110	OP056144	SRR21430353	MZFC	Villa Nueva, 800 m al SEuphonia Veracruz. Mexico.	19.7699444	-96.862083
Euphonia hirundinacea hirundinacea	TXT-27	XXX	SRR21430349	MZFC	Ejido La Perla de San Martín, Campamento B. Veracruz. Mexico.	18.55125	-95.123667
Euphonia hirundinacea hirundinacea	TXT-111	OP056145	XXX	MZFC	Ejido La Perla de San Martín, Campamento B. Veracruz. Mexico.	18.55125	-95.123667
Euphonia imitans	B71975	OP056160	SRR21430376	LSUMZ	Osa, Reserva Forestal Golfo Dulce, Rd. to DrakEuphonia Puntaarenas. Costa Rica.	8.672028	83.505194
Euphonia jamaica	33115	OP056128	SRR21430450	FMNH	Portland, Hollywell Park. Surrey. Jamaica.	18.085726	-76.732169
Euphonia jamaica	33116	OP056129	SRR21430449	FMNH	Trelawny, Good HopEuphonia Cornwall. Jamaica.	18.4295736	-77.691852
Euphonia laniirostris crassirostris	B28567	OP056146	SRR21430367	LSUMZ	Achiote Road at Rio Providencia. Colon ProvincEuphonia Panama.	9.2284121	-80.031107
Euphonia laniirostris	390050	OP056147	SRR21430440	FMNH	Cachoeira Nazare, W bank Rio Jiparana. Rondônia. Brasil.	-9.7333333	-61.883333
Euphonia laniirostris	474504	OP056148	SRR21430343	FMNH	Rio Verde Amazonas. Peru.	-6.7217	-77.425
Euphonia luteicapilla	B16078	XXX	SRR21430386	LSUMZ	Rio Copey, ca 4 km E Jaco. Puntaarenas. Costa Rica.	9.646392	-84.604991
Euphonia luteicapilla	B28423	OP056136	SRR21430373	LSUMZ	0.5 km SSW mouth Rio Farfan. Panama ProvincEuphonia Panamá.	8.926338	-79.56553
Euphonia luteicapilla	B28424	OP056138	SRR21430369	LSUMZ	0.5 km SSW mouth Rio Farfan. Panama ProvincEuphonia Panamá.	8.926338	-79.56553
Euphonia luteicapilla	B28445	OP056137	SRR21430368	LSUMZ	1 1/4 mi. SW Cerro Farfan. Panama ProvincEuphonia Panamá.	9.0637694	-79.661071
Euphonia mesochrysa	474505	OP056158	SRR21430342	FMNH	1200m of Río VerdEuphonia Amazonas. Perú	-6.7217	-77.425

Euphonia minuta	B9714	XXX	SRR21430370	LSUMZ	Nicolás Suárez, 12 km S by road of Cobija, 8 km W by road of Mucden. Pando Deparment. Bolivia.	-11.183	-68.967
Euphonia minuta	390051	OP056142	SRR21430439	FMNH	Cachoeira Nazare, W bank Rio Jiparana. Rondônia. Brasil.	-9.75	-61.916667
Euphonia minuta minuta	474508	OP056143	SRR21430341	FMNH	Río Verde Amazonas. Peru.	-6.7217	-77.425
Euphonia pectoralis	427269	XXX	SRR21430362	FMNH	Ibateouara, Envenho Ceimba, Usina Serra GrandEuphonia Alagoas. Brasil.	-8.9759411	-36.058886
Euphonia pectoralis	427270	OP056155	SRR21430361	FMNH	Ibateouara, Envenho Ceimba, Usina Serra GrandEuphonia Alagoas. Brasil.	-8.9759411	-36.058886
Euphonia plumbea	21372/187871	OP056131	SRR21430430	ANSP	Iwokrama Reserve; Iwokrama-Kurupukai Base Camp. Potaro-Siparuni. Brasil.	4.28333333	-58.516667
Euphonia plumbea	B25459	OP056132	SRR21430395	LSUMZ	Igarape da Arraia, ca --km N Barcelos. Amazonas. Brasil.	-6.9107034	-59.130888
Euphonia rufiventris	457595	OP056156	SRR21430345	FMNH	Japura, Río Mapari. Amazonas. Brasil.	-2.04972	-67.2631
Euphonia rufiventris	B1176	OP056157	SRR21430392	LSUMZ	Río Beni, ca 20 km por el río N. Puerto Linares. La Paz. Bolivia.	-15.4024329	-67.583965
Euphonia saturata	B67621	OP056130	SRR21430377	LSUMZ	La Coja. Department Tumbes. Peru.	-3.600278	-80.206667
Euphonia violacea rodwayi	B35986	OP056152	SRR21430383	LSUMZ	1.5 km W Summit Morne Bleu. St. George County. Republic of Trinidad and Tobago.	10.7162533	-61.282825
Euphonia violacea auranticollis	427255	OP056149	SRR21430437	FMNH	Taquaritinga. Pernambuco. Brasil.	-7.8376347	-36.017326
Euphonia violacea auranticollis	427264	OP056150	SRR21430436	FMNH	Ibateouara, Envenho Ceimba, Usina Serra GrandEuphonia Alagoas. Brasil.	-8.9759411	-36.058886
Euphonia violacea violacea	21748/187863	OP056151	XXX	ANSP	Iwokrama Reserve; Turtle Mountain; Paddle Rock Camp at Essequibo River,	4.28333333	-58.516667

					4.7 mi NW Kurupukari. Potaro-Siparuni. Guayana.		
Euphonia xanthogaster	B10698	OP056168	SRR21430401	LSUMZ	W. bank Rio Shesha, 65 km ENE Pucallpa. Ucayali Department. Peru.	-8.1055773	-74.521755
Euphonia xanthogaster	B21214	OP056167	SRR21430398	LSUMZ	Ridge E junction of Rio Tavara-Guacamayo-Condamo. Puno Department. Peru.	-13.5047	-69.6844
Euphonia xanthogaster chocoensis	B2157	OP056166	SRR21430397	LSUMZ	About 6 km NW Cana. Darien ProvincEuphonia Panama.	7.8277396	-77.752302
Euphonia xanthogaster ruficeps	B39186	OP056169	SRR21430380	LSUMZ	Provincia de Chapare, San Onofre, ca. 43 km al W de Villa Tunari. Cochabamba Department. Bolivia.	-17.146389S	-65.779444
Euphonia xanthogaster ruficeps	B41775	XXX	SRR21430448	LSUMZ	Prov. Chapare San Onofre, ca 43 km W Villa Tumari. Cochabamba Department. Bolivia.	-16.8536111	-64.220556
Cocothrauthes abeillei	JCN046	XXX	SRR21430355	MZFC	Mexico.		
Cocothrauthes abeillei	RAY11-023	XXX	SRR21430351	MZFC	Mexico.		
Haemorhous mexicanus	CPM357	XXX	SRR21430358	MZFC	Mexico.		
Fringilla coelebs	16_129	XXX	SRR21430360	CSIC	Río Moros, Valdeprados. Segovia. Spain.	40.81017	-4.26463
Fringilla coelebs	DOT-20961	XXX	SRR21430357	AMNH	Hössjö. Sweden.		
Fringilla montifringilla	DOT-20978	XXX	SRR21430356	AMNH	Lulea Lappmark; Luovauren. Sweden.		
Rhodinocichla rosea	URRA-4120	XXX	SRR21430348	MZFC	Mexico.		
LSUMN: Lousiana Museum of Natural History. FMNH: Field Museum of Natural History. ANSP: The Academy of Natural Sciences of Drexel University. KU: Kansas University. MZFC: Museo de Zoología de la Facultad de Ciencias. AMNH: American Museum of Natural History. CSIC: Consejo Superior de Investigaciones Científicas.							

**Table S2.** ND2 sequences obtained from Genbank	
Specie	Genbank Number Accession
E. affinis	MT452146
E. affinis	MT452148
E. affinis	MT452149
E. affinis	MT452151
E. affinis	MT063173
E. godmani	MT452159
E. godmani	MT452167
E. godmani	MT452164
E. jamaica	MT063167
Chlorophonia occipitalis	EF529844
Haemothrous mexicanus	KC292816
Coccothrautes coccothrautes	KX109684
Fringilla coelebs	KF672966

Table S3. Results after filtering and clustering nextRAD data from Euphoniinae samples and outgroup samples.

IDSample	Reads raw	Reads passed filter	Clusters total	Clusters hidepth	Hetero est	Error est	Reads consens	Loci in assembly
1983	4205599	4143186	317372	71305	0.00812	0.00162	67838	2008
2099	4258125	4198223	226138	60291	0.00616	0.00125	58319	1860
3230	4178389	4118334	355326	69424	0.00914	0.00198	66080	2009
9346	4266696	4201546	250108	68027	0.00566	0.00128	65918	1828
33115	4900611	4833939	362356	82489	0.00647	0.00169	78920	2135
33116	4568958	4514245	262217	66657	0.00509	0.00131	64540	2017
88442	4536907	4484856	231531	66087	0.0034	0.00102	64685	1992
91216	4924394	4864123	289095	77347	0.00425	0.00117	75243	2020
95132	3901199	3851394	175879	52406	0.00312	0.000923	51337	1590
95133	2374359	2343538	123826	32757	0.00316	0.000977	32085	1179
389276	4318717	4249925	260899	68124	0.00639	0.00142	65624	2038
390049	5654665	5575804	367440	84011	0.0086	0.00154	80394	2149
390050	3523507	3469469	343724	66978	0.00762	0.00179	63464	1812
390051	4035036	3989757	188515	52114	0.005	0.00111	50752	1760
392636	4397393	4329269	274329	72099	0.0063	0.00138	69786	1937
427255	2169987	2141479	122241	33924	0.00401	0.00106	33168	1114
427264	2968153	2931045	188755	48136	0.0041	0.00107	46970	1387
427269	3177787	3143157	181998	50326	0.00452	0.00114	48963	1595
427270	3741597	3701651	194930	57341	0.00418	0.00111	55861	1644
433910	4450795	4400248	247569	69360	0.00451	0.00113	67599	1922
457595	5398586	5332787	275303	83467	0.00599	0.00126	80929	1929
474504	5076052	5020043	246306	75879	0.00437	0.00107	74256	2014
474505	6320700	6242114	280123	86059	0.00479	0.00115	83808	2031
474508	5078254	5015112	234988	74674	0.00533	0.0012	72562	1843
474515	3784411	3734173	322251	68772	0.00605	0.00157	66366	2060

08-807	2285743	2255033	123121	33087	0.00465	0.00113	32330	1259
16_129	3939986	3898704	185140	54654	0.0065	0.00115	52989	1181
21372/187871	4825430	4749194	285079	83891	0.00606	0.00133	80973	1908
21423/187874	5616099	5529202	385646	97103	0.007	0.00173	93075	2165
21424/187875	5456079	5380655	275972	81491	0.00541	0.00126	78995	2004
21748/187863	4233765	4185615	223835	67786	0.00443	0.00105	66269	1852
21809/187876	4577905	4519003	227545	63479	0.00533	0.0011	61544	1633
90102/3199	4640528	4572701	275428	69030	0.0066	0.00139	66385	1933
90311/3226	2821082	2780691	149456	37311	0.00481	0.00108	36367	1060
B10698	2883413	2851566	175836	47462	0.00533	0.00112	45911	1433
B11319	5744319	5667432	283664	83719	0.0041	0.00117	81278	1955
B11337	5123415	5060693	247715	74051	0.00346	0.00106	72260	2012
B1176	4449971	4398644	226038	64687	0.00634	0.00122	62210	1726
B11778	3168193	3104168	301130	61179	0.00696	0.00156	58344	1666
B12361	1386894	1367535	93867	21044	0.00358	0.000993	20646	761
B12875	4694325	4639177	302876	83181	0.00545	0.00144	80398	2054
B15102	3809918	3761089	221302	58634	0.00583	0.00124	56836	1772
B16031	3796848	3750177	172308	51997	0.00459	0.00112	50793	1739
B16078	2857687	2822104	152308	40930	0.00415	0.00112	39880	1429
B19863	5333687	5268294	359086	91304	0.00522	0.00142	88052	2155
B21214	3132282	3081574	315880	61708	0.00758	0.00192	58571	1739
B2157	3667875	3624463	213614	56934	0.00595	0.00133	55067	1832
B25459	4032323	3983455	197254	58553	0.0046	0.00111	57043	1687
B26491	2332756	2297583	134194	31889	0.0042	0.000997	31072	963
B27273	3968578	3906182	375482	72925	0.0076	0.00188	69133	2078
B27826	2977912	2940086	197025	47061	0.00584	0.00139	45317	1595
B28567	2373520	2346832	121956	33237	0.00387	0.001	32490	1209

B28423	4002173	3947895	192211	51585	0.00509	0.00119	50039	1731
B28424	3923068	3874060	176488	50859	0.00428	0.000963	49631	1523
B28445	4322866	4265651	205182	60084	0.0047	0.0012	58362	1761
B28780	4368688	4312457	212724	56967	0.00532	0.00116	55422	1855
B30015	4443982	4385860	312254	73021	0.00657	0.00146	70124	2125
B32512	3196512	3137649	308427	50955	0.00859	0.00208	47861	1793
B33430	3505995	3449859	175698	45900	0.00512	0.00111	44460	1423
B35986	3348159	3309273	167714	48797	0.00435	0.00103	47771	1599
B38380	3854525	3803017	346108	71174	0.00686	0.00177	68055	2017
B38489	3805422	3760834	247590	67139	0.00497	0.00135	65129	1929
B39186	2069383	2043864	136655	33905	0.00354	0.00104	33209	1224
B41775	2854627	2819993	148971	38947	0.00392	0.000986	37950	1220
B5592	4882168	4821193	200313	61358	0.00402	0.000973	59939	1681
B6223	5120032	5053844	319127	75371	0.00678	0.0015	71857	1947
B67621	4239216	4181278	195317	58582	0.00365	0.00104	57264	1747
B71975	3416688	3368595	163903	47296	0.0042	0.00104	46108	1635
B72152	4219672	4166383	273446	75218	0.00505	0.00122	73087	1863
B72212	4538732	4474881	367582	78973	0.00672	0.00168	75663	2096
B72236	5260737	5194178	255628	72852	0.0061	0.00118	70590	1994
B72245	3945739	3894853	258948	59389	0.00775	0.00162	56371	1778
B9714	3286330	3250123	138120	40167	0.00399	0.00099	39291	1497
Conacyt-1301	3966691	3879358	392029	57317	0.0153	0.00342	50926	1421
CPM-357	3690594	3639870	293598	70035	0.00736	0.00171	67022	1210
DOT-20961	3123792	3067188	407505	48388	0.0176	0.00366	42171	1062
DOT-20978	4602335	4550697	328392	76572	0.0105	0.00187	72310	1366
JCN046	2590490	2547501	311250	44366	0.00954	0.00264	41290	777
MISA-110	2745867	2705360	339357	57413	0.00854	0.00212	53879	1370

MISA-68	3792086	3748642	312309	74422	0.0044	0.0015	71911	1792
ORT-03	3631565	3562989	376882	63368	0.00859	0.00239	59158	1896
RAY11-023	2753746	2720415	243843	44765	0.00844	0.0023	41871	830
TXT-27	1040017	1016414	207777	12920	0.0155	0.00257	11377	311
URRA-4120	4708277	4651936	318216	85497	0.0052	0.00144	82452	1190
VGR-1073	3388042	3328968	366642	63759	0.00744	0.00219	60363	1849
VGR-1189	3203998	3129131	448566	47268	0.0143	0.00389	41121	1635

Table S4. Sample coverage for nextRAD data from Euphoniinae samples and outgroup samples.

ID sample	Sample coverage	ID sample	Sample coverage
**TXT-27**	311	**B72245**	1778
**B12361**	761	**MISA-110**	1792
**JCN046**	777	**B32512**	1793
**RAY11-023**	830	**390050**	1812
**B26491**	963	**21739**	1814
**90102/3199**	1060	**9346**	1828
**DOT-20961**	1062	**B2157**	1832
**427255**	1114	**474508**	1843
**95133**	1179	**VGR-1073**	1849
**16_129**	1181	**21748/187863**	1852
**URRA-4120**	1190	**B28780**	1855
**B28567**	1209	**2099**	1860
**CPM357**	1210	**B72152**	1863
**B41775**	1220	**ORT-003**	1896
**B39186**	1224	**21372/187871**	1908

**DOT-20978**	1366	**433910**	1922
**MISA-68**	1370	**457595**	1929
**427264**	1387	**B38489**	1929
**Conacyt-1301**	1421	**3230**	1933
**B33430**	1423	**392636**	1937
**B16078**	1429	**B6223**	1947
**B10698**	1433	**B11319**	1955
**B9714**	1497	**88442**	1992
**B28424**	1523	**B72236**	1994
**95132**	1590	**21424/187875**	2004
**427269**	1595	**1983**	2008
**B27826**	1595	**90311/3226**	2009
**B35986**	1599	**B11337**	2012
**21809/187876**	1633	**474504**	2014
**B71975**	1635	**33116**	2017
**VGR-1189**	1635	**B38380**	2017
**427270**	1644	**91216**	2020
**B11778**	1666	**474505**	2031
**B5592**	1681	**389276**	2038
**B25459**	1687	**B12875**	2054
**B1176**	1726	**474515**	2060
**B28423**	1731	**B27273**	2078
**B16031**	1739	**B72212**	2096
**B21214**	1739	**B30015**	2125
**B67621**	1747	**33115**	2135
**390051**	1760	**390049**	2149
**B28445**	1761	**B19863**	2155

**B15102**	1772	**21423/187874**	2165

Table S5. Substitution models for nucleotide data partitions selected using the BIC in PartitionFinder.

Subset	Best Model	Sites
1	GTR+I+G+X	59654
2	GTR+I+G+X	43648
3	GTR+I+G+X	15551
4	GTR+G+X	3819
5	GTR+G+X	10749
6	GTR+G+X	12386
7	GTR+G+X	3444
8	GTR+G+X	29736
9	GTR+G+X	8350
10	GTR+I+G+X	13510
11	GTR+I+G+X	5676
12	GTR+G+X	7949
13	GTR+I+G+X	3855
14	GTR+G+X	3811
15	GTR+I+G+X	26387
16	GTR+G+X	13427
17	GTR+I+G+X	26247
18	GTR+G+X	7829
19	GTR+G+X	10450
20	GTR+I+G+X	6920
21	GTR+I+G+X	32533
22	GTR	1219
23	GTR+I+G+X	9485
24	GTR+I+G+X	5541
25	GTR+I+G+X	4090

26	GTR+I+G+X	549
27	GTR+I+G+X	1197
28	GTR+I+G+X	858
29	GTR+I+G+X	141

Laboratory process and sequence preparation for nextRAD sequencing and quality filtering and Denovo alignment results.

- **Text S1. Laboratory process and sequence preparation for nextRAD sequencing.**

We extracted total genomic DNA from the tissue samples using the DNeasy tissues kit (Qiagen, Valencia, CA, USA) or the phenol: chloroform protocol (Hillis et al. 1996). The quality of DNA extractions was verified using gel electrophoresis. The DNA concentration was determined with a Qubit 3 fluorometer (ThermoFisher). The RAD sequence data was obtained using the nextRAD protocol by the company SNPsaurus (http://snpsaurus.com/) . We sent at least 50 ng of clean DNA at a concentration of ~5.0-10.0 ng/ul (maximum of 25 ng/ul). Genomic DNA was converted to nextRAD genotypes using sequencing libraries (SNPsaurus, LLC) as in Russello et al. (2015). Genomic DNA was first fragmented with Nextera Flex reagent (Illumina, Inc), which also ligates short adapter sequences to the ends of the fragments. The Nextera reaction was scaled for fragmenting 20 ng of genomic DNA. Fragmented DNA was then amplified for 27 cycles at 74 degrees, with one of the primers matching the adapter and extending 10 nucleotides into the genomic DNA with the selective sequence GTGTAGAGCC. Thus, only fragments starting with a sequence that could be hybridized by the selective sequence of the primer were efficiently amplified. The nextRAD libraries were sequenced on a single lane of an Illumina HiSeq 4000 with a single-end 150 bp protocol (University of Oregon).

- **Text S2. Quality filtering and Denovo alignment results.**

We found a strong relationship between the missing data and phylogenetic distances, with the highest values of Pearson correlations. The heatmap in figure 1 shows the threshold value test for these six clusters in which Pearson's correlation coefficient is less than 0.87. We found a strong relationship between the missing data and phylogenetic distances for 0.88 to 0.90 clust threshold values and the lowest correlation with 0.87 (Figure 2). This suggests that at 0.88, the most divergent alleles oversplit from their loci, which increases missing data. The graphs of the mean bootstrap values did not indicate significant differences between the clust threshold value. However, for phylogenies with only 10% of missing loci, the mean bootstrap values show more variation. The first 3 PCs' cumulative variance decreased from 0.85 to 0.90 clust value, with a breakpoint at 0.87 and a rapid decline beginning at 0.88 (3). This pattern suggests that as we increase the clustering value, the alignment variance decreases, probably due to the presence of more homozygous loci. For the fourth metric, we found a faster increase in total loci and total SNPs from 0.88 to 0.9 clust value (4 and 5). While heterozygous mean and percent of heterozygous sites decreased when increasing from 0.88 to 0.9, the highest rate of heterozygous loci was at a clust threshold value of 0.85 (6 and 7). The goal of optimizing a de novo alignment for Rad seq data is to minimize the

Laboratory process and sequence preparation for nextRAD sequencing and quality filtering and Denovo alignment results.

presence of paralogous loci. This requires a balance between the total loci and heterozygosity. Low clustering values tend to include more paralogs in the alignment, which increases heterozygosity and decreases the number of loci. Meanwhile, higher clustering values drop the most divergent alleles as loci, which increases the missing data and decreases heterozygosity. Under this reasoning and based on the metric values, we set 0.87 as the optimal clustering for this alignment. The final alignment recovered 2,570 loci, 369,000 pb, and 37.43 % missing sites. The total SNPs recovered are 57, 575 and 37.26% missing sites.

1. Heat maps show pairwise data missingness at clustering thresholds of 0.85 (a), 0.86 (b), 0.87 (c), 0.88 (d), 0.89 (e) and 0.90 (f).

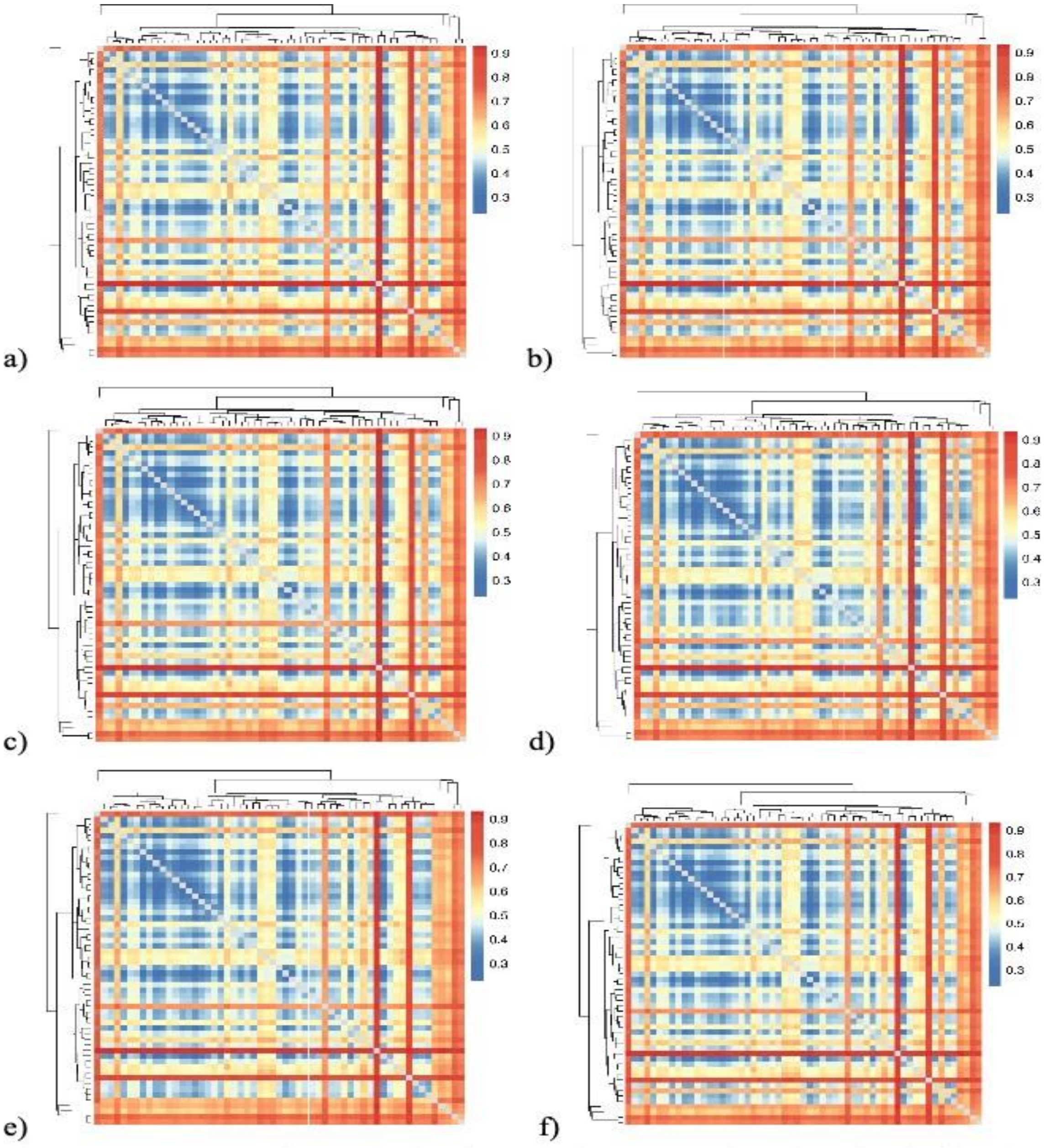

Laboratory process and sequence preparation for nextRAD sequencing and quality filtering and Denovo alignment results.

2. Pearson correlation between genetic distance and missingness.

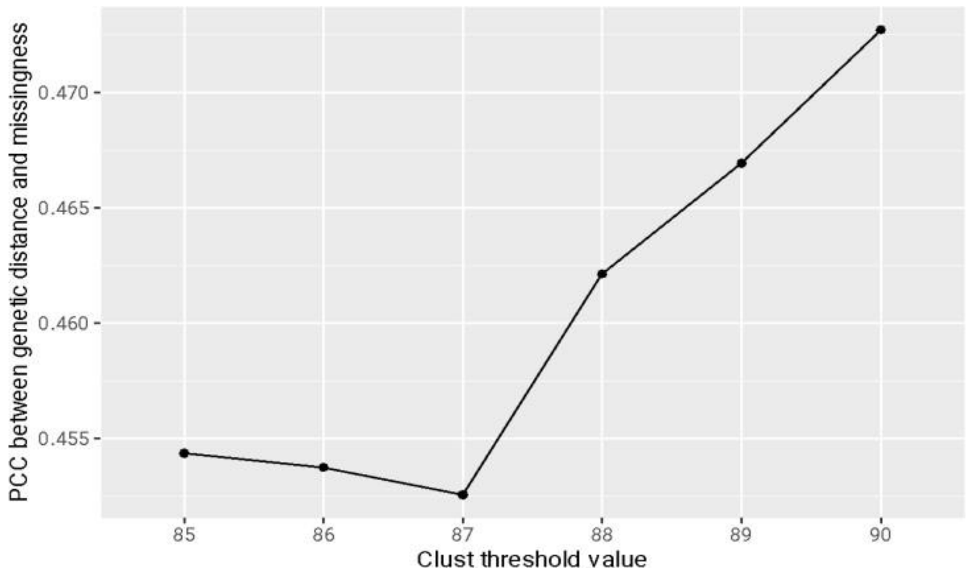

3. Cumulative variance in first 3 PCs.

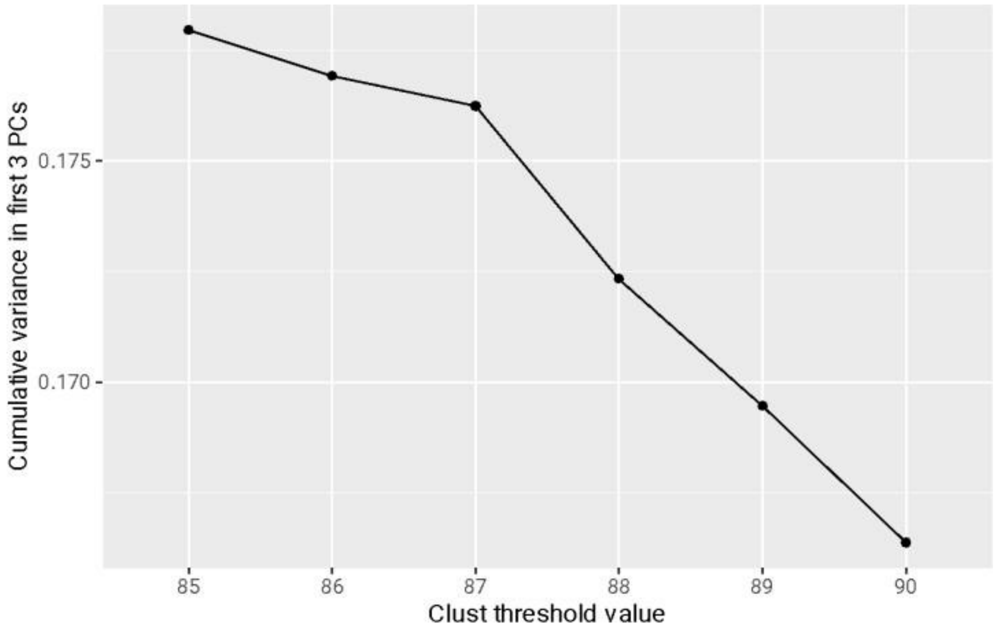

Laboratory process and sequence preparation for nextRAD sequencing and quality filtering and Denovo alignment results.

4. Total SNPs by clust value.

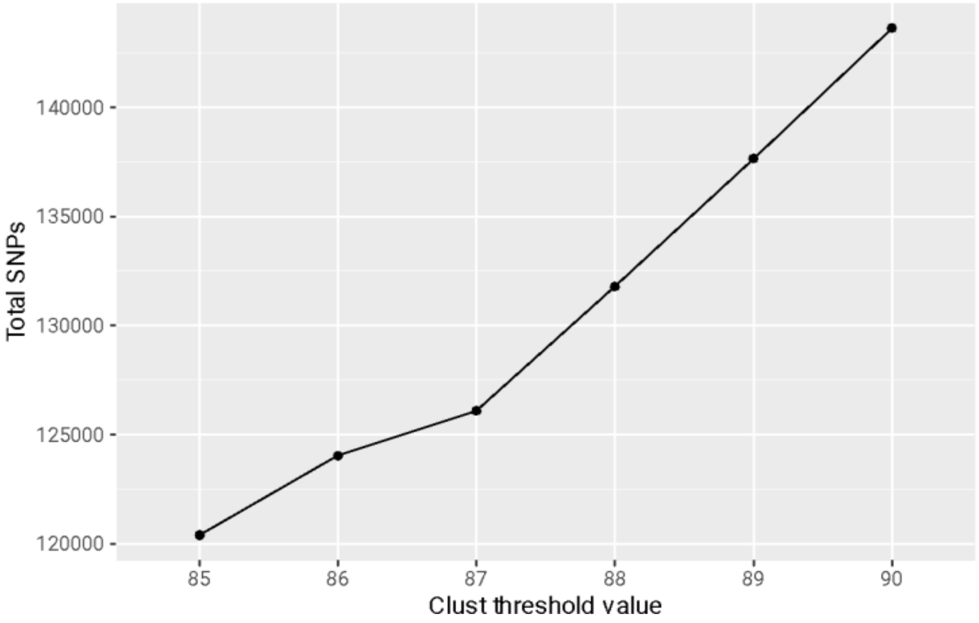

5. Total loci by clust value.

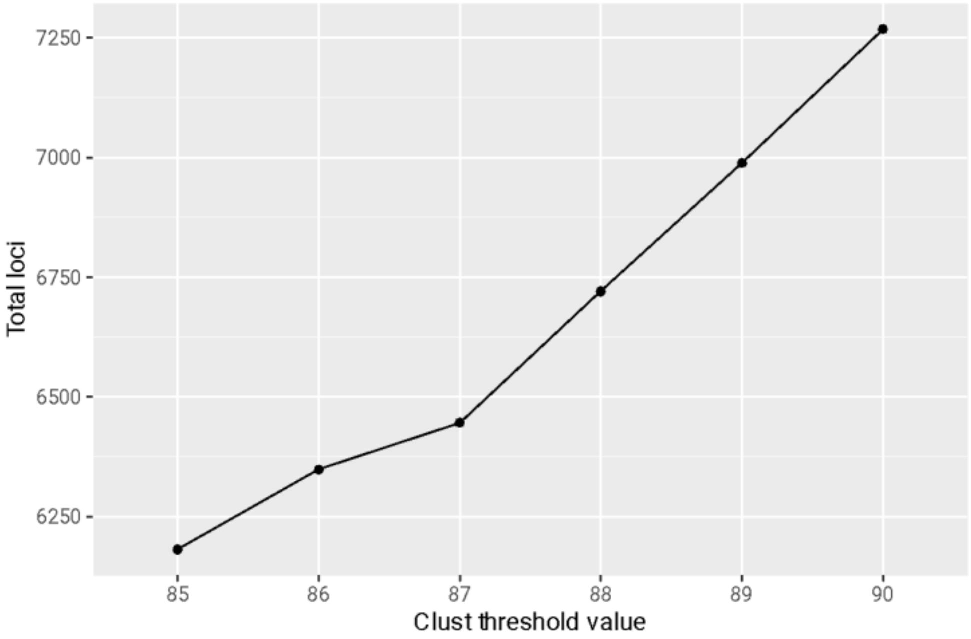

Laboratory process and sequence preparation for nextRAD sequencing and quality filtering and Denovo alignment results.

6. Heterozygosis mean.

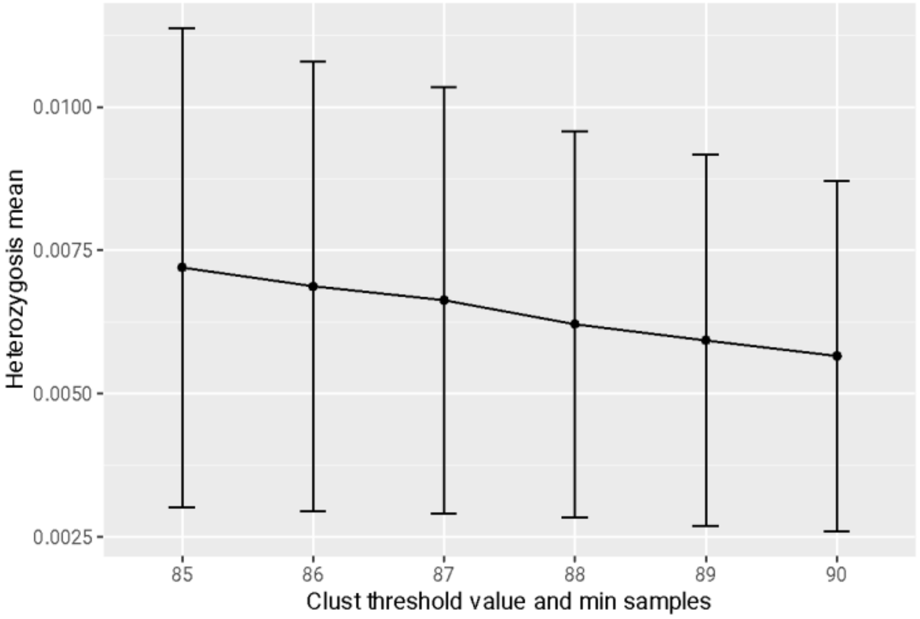

7. Heterozygosis.

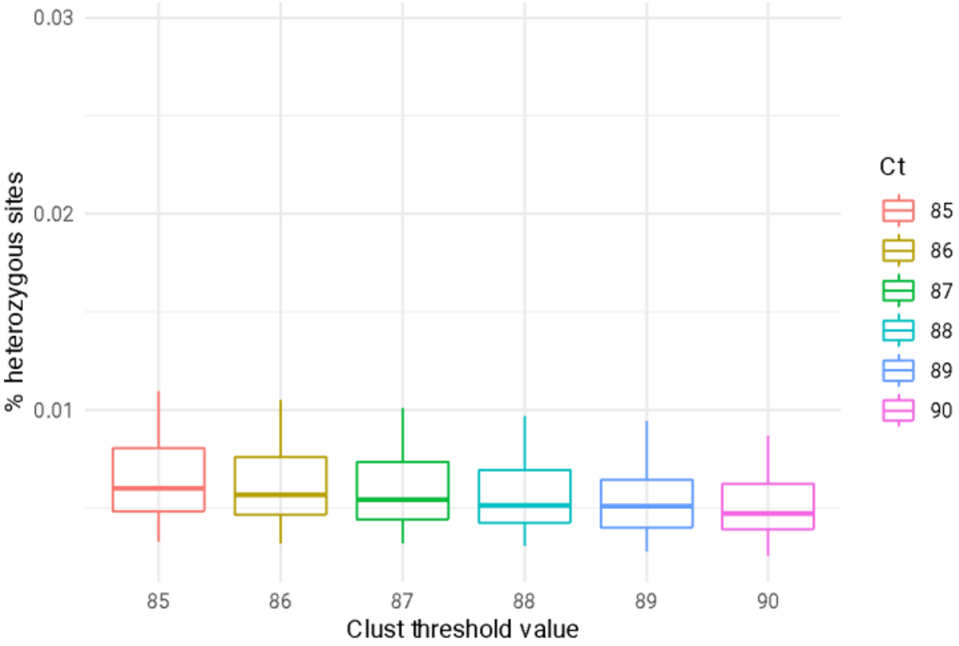

Outputs of BioGeoBEARS analysis

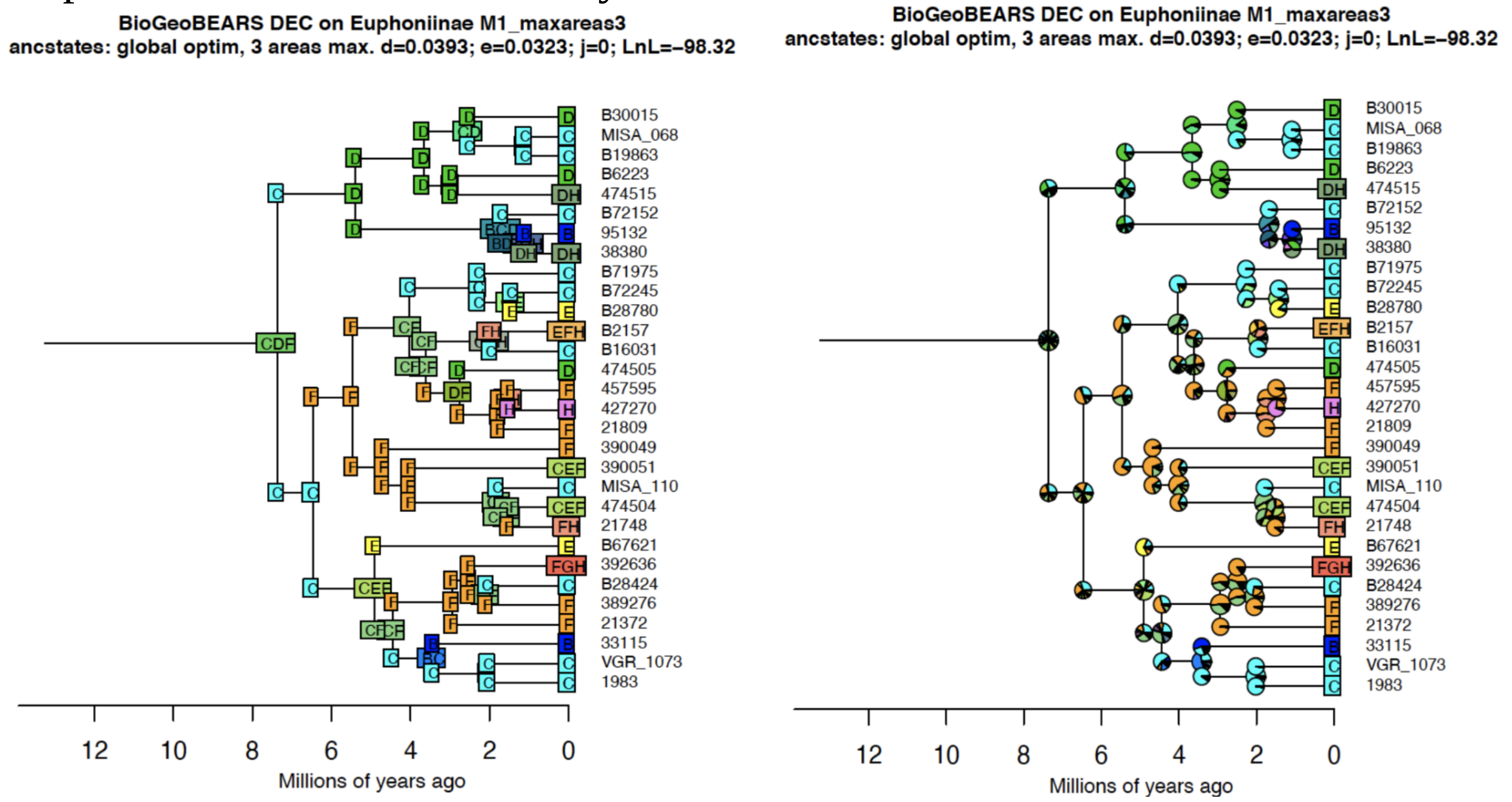

Figure S1. DEC_maxareas3_v1 B=Caribbean C=Mesoamerica D=Andes E=Pacific F=Amazonas G=Chacoan H=Paraná-Atlantic Forest.

Outputs of BioGeoBEARS analysis

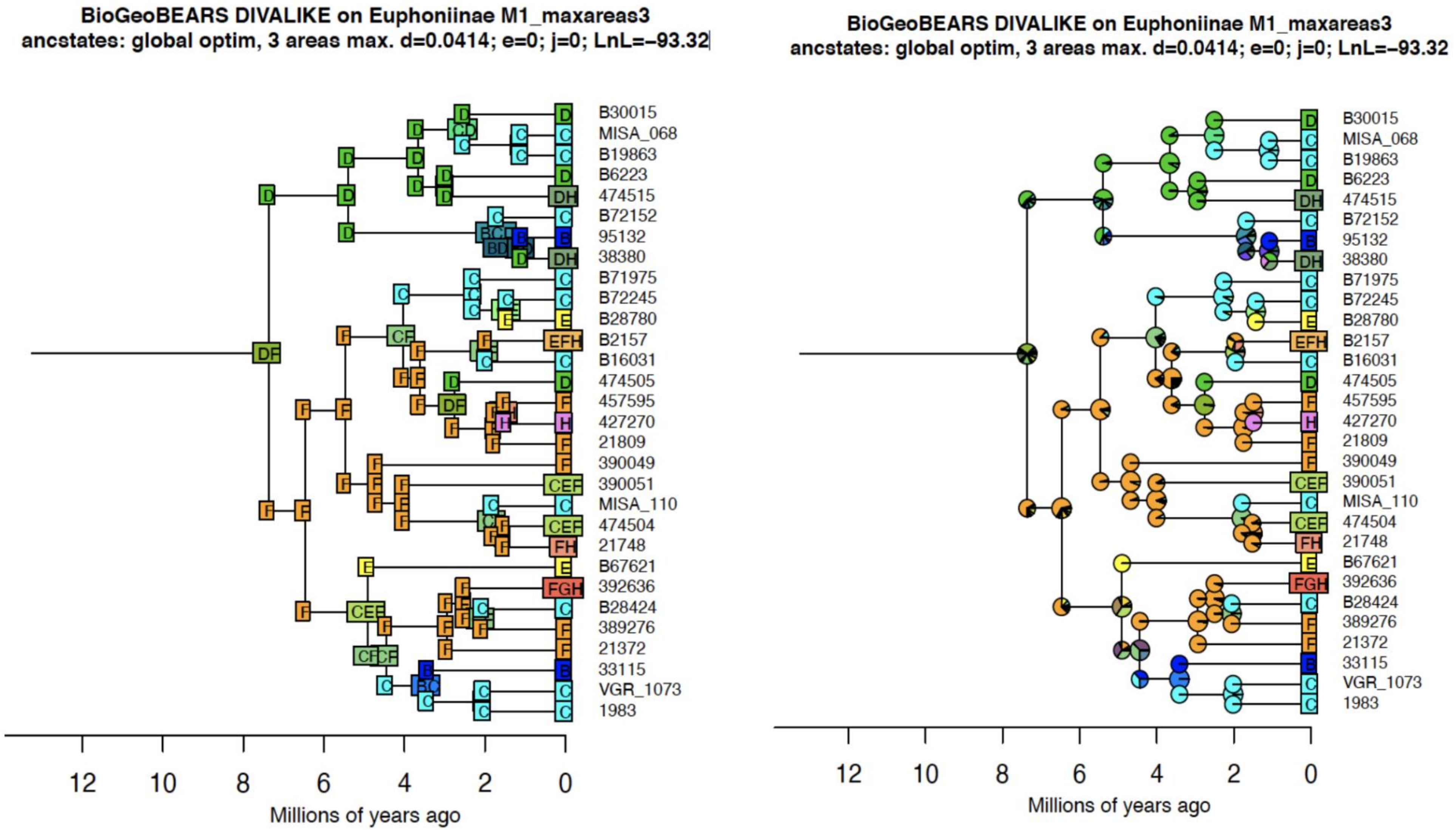

Figure S2. DIVALIKE_maxareas3_v1 B=Caribbean C=Mesoamerica D=Andes E=Pacific F=Amazonas G=Chacoan H=Paraná-Atlantic Forest.

Outputs of BioGeoBEARS analysis

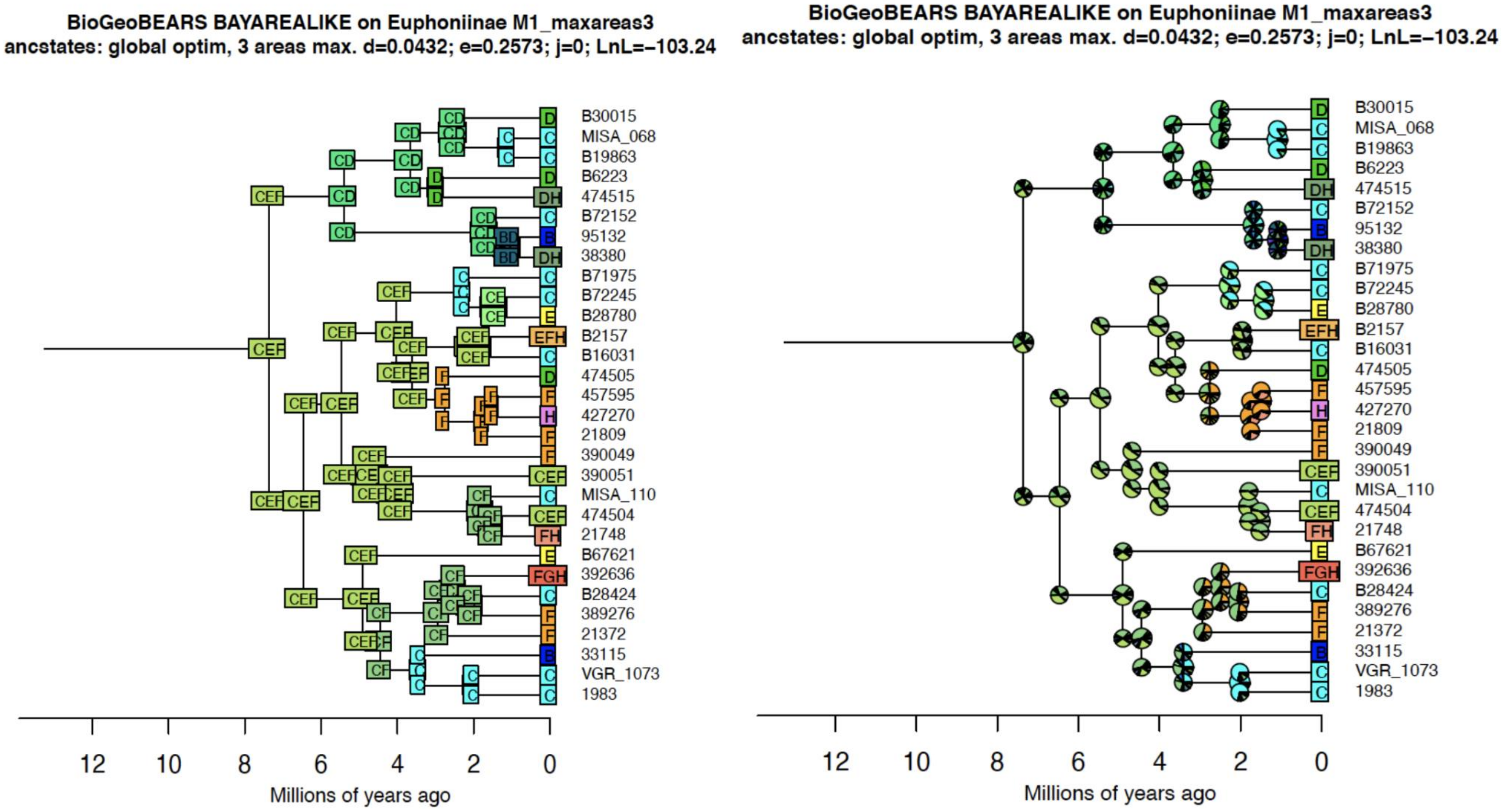

Figure S3. BAYAREALIKE_maxareas3_v1 B=Caribbean C=Mesoamerica D=Andes E=Pacific F=Amazonas G=Chacoan H=Paraná-Atlantic Forest.
